# Rare, long‐distance dispersal underpins genetic connectivity in the pink sea fan, *Eunicella verrucosa*

**DOI:** 10.1111/eva.13649

**Published:** 2024-03-07

**Authors:** Kirsty L. Macleod, Tom L. Jenkins, Matthew J. Witt, Jamie R. Stevens

**Affiliations:** ^1^ Department of Biosciences, Faculty of Health and Life Sciences University of Exeter Exeter UK

**Keywords:** genetic structure, oceanographic modelling, octocoral, RAD sequencing, single nucleotide polymorphism

## Abstract

Characterizing patterns of genetic connectivity in marine species is of critical importance given the anthropogenic pressures placed on the marine environment. For sessile species, population connectivity can be shaped by many processes, such as pelagic larval duration, oceanographic boundaries and currents. This study combines restriction‐site associated DNA sequencing (RADseq) and passive particle dispersal modelling to delineate patterns of population connectivity in the pink sea fan, *Eunicella verrucosa*, a temperate octocoral. Individuals were sampled from 20 sites covering most of the species' northeast Atlantic range, and a site in the northwest Mediterranean Sea to inform on connectivity across the Atlantic‐Mediterranean transition. Using 7510 neutral SNPs, a geographic cline of genetic clusters was detected, partitioning into Ireland, Britain, France, Spain (Atlantic), and Portugal and Spain (Mediterranean). Evidence of significant inbreeding was detected at all sites, a finding not detected in a previous study of this species based on microsatellite loci. Genetic connectivity was characterized by an isolation by distance pattern (IBD) (*r* ^2^ = 0.78, *p* < 0.001), which persisted across the Mediterranean‐Atlantic boundary. In contrast, exploration of ancestral population assignment using the program ADMIXTURE indicated genetic partitioning across the Bay of Biscay, which we suggest represents a natural break in the species' range, possibly linked to a lack of suitable habitat. As the pelagic larval duration (PLD) is unknown, passive particle dispersal simulations were run for 14 and 21 days. For both modelled PLDs, inter‐annual variations in particle trajectories suggested that in a long‐lived, sessile species, range‐wide IBD is driven by rare, longer dispersal events that act to maintain gene flow. These results suggest that oceanographic patterns may facilitate range‐wide stepping‐stone genetic connectivity in *E. verrucosa* and highlight that both oceanography and natural breaks in a species' range should be considered in the designation of ecologically coherent MPA networks.

## 1 INTRODUCTION

Marine connectivity, the extent to which populations across a species' range are linked by the movement of gametes, larvae, juvenile or adults (Palumbi, 2003), is integral to population dynamics in marine systems (Cowen & Sponaugle, 2009). Isolated populations, for example, may rely on immigration from adjacent or distant populations to maintain a stable population size (demographic connectivity), or to introduce genetic diversity into a population via gene flow (genetic connectivity), which can mitigate the effects of genetic drift and inbreeding (Gagnaire et al., 2015; Lowe & Allendorf, 2010). The degree of genetic connectivity among benthic marine species' populations varies and can depend on the length of time that a species' larvae remain viable in the water column, a period known as the Pelagic Larval Duration (PLD) (Ellis et al., 2023; Weersing & Toonen, 2009). In addition, a myriad of other factors including evolutionary history (such as re‐establishment from post‐glacial refugia (Jenkins, Castilho, et al., 2018), oceanographic barriers (Johannesson et al., 2018; Patarnello et al., 2007) and selection (Benestan et al., 2016; Lehnert et al., 2018) can shape genetic structure within marine taxa. Given the scale of anthropogenic threats facing marine ecosystems, including localised pressures such as habitat degradation via bottom trawling, and more wide‐ranging threats associated with climate change (Gazulla et al., 2021; Pivotto et al., 2015), it is important to understand patterns of marine connectivity and diversity and how they might affect future population persistence (Griffiths et al., 2020; Torrents et al., 2008).

For sessile benthic species, such as the cold‐water soft corals (Malacalcyonacea, formerly Alcyonacea; see McFadden et al., 2022), population connectivity is dependent on the dispersal and exchange of a pelagic larval stage among populations, which can be influenced by hydrographic conditions, such as oceanic currents (Cowen & Sponaugle, 2009) and environmental gradients (e.g. temperature, Lehnert et al., 2018), and biological traits of larvae (e.g. swimming behaviour, Martínez‐Quintana et al., 2015). Factors shaping fine‐scale (<1 km) and broad‐scale genetic connectivity in cold‐water soft corals include reproductive ecology (broadcast spawner or brooder) (Aurelle et al., 2018; Holland et al., 2017), PLD (Mokhtar‐Jamaï et al., 2011) and oceanic fronts (Aurelle et al., 2011), while local conditions, such as elevated sea temperature (Garrabou et al., 2022) and disease outbreaks (Hall‐Spencer et al., 2007), can affect population demography.

*Eunicella verrucosa* is a soft coral species distributed across the northeast Atlantic from western Ireland to, reportedly, the coast of Mauritania in West Africa (IUCN, 2017; Hayward & Ryland, 1995) and as far as the Aegean Sea in the eastern Mediterranean Sea (Chimienti et al., 2020). In the northeast Atlantic, *E. verrucosa* is typically found in depths shallower than 50 m (Readman & Hiscock, 2017) and can occur in dense, ‘forest‐like’ (Chimienti et al., 2020) aggregations, such as those described in southern UK coastal waters (Jenkins & Stevens, 2022), while in the Mediterranean Sea it is present down to depths of up to 200 m (Grasshoff, 1992; Sartoretto & Francour, 2012). Typically, soft corals exhibit patchy distributions (Grasshoff, 1992; Poliseno et al., 2022), driven by habitat suitability (Gori et al., 2011), abiotic factors (irradiance and hydrography, Linares, Coma, et al., 2008) and biological interactions, such as competition with algae (Cúrdia et al., 2013). This patchy pattern of distribution appears particularly characteristic of pink sea fan populations at their range‐edges, for example, in western Ireland and in Pembrokeshire, southwest Wales (Holland et al., 2017).

The pink sea fan can act as a key ecological substrate for various epifauna, increasing the structural complexity of benthic ecosystems (Pikesley et al., 2016; Wood, 2013). For *E. verrucosa*, this species' slow‐growth (Coz et al., 2012), longevity (>50 years old) (Wood, 2013) and physical three‐dimensional structure can render local populations vulnerable to ecological pressures, including physical disturbance (Readman & Hiscock, 2017) and disease (Hall‐Spencer et al., 2007). As a result, this species is IUCN listed and used as a ‘protected feature’ for the designation of Marine Protected Areas (MPAs) in England and Wales.

The first dedicated studies investigating genetic connectivity in this species were conducted by Holland et al. (2013, 2017) using 13 microsatellite loci and detected three distinct genetic clusters: northwest Ireland, southwest Britain/northern France and southern Portugal. While a moderate signal of isolation by distance (IBD) was detected, incomplete sampling coverage limited estimates of connectivity throughout the pink sea fan's northeast Atlantic distribution. Moreover, potential dispersal between oceanic basins (Mediterranean Sea/northeast Atlantic) was not studied. Conversely, the same study found minimal geographic genetic structuring in an octocoral, *Alcyonium digitatum*, possibly due to its spawning period occurring later in the year, which may facilitate longer dispersal distances via wind‐driven currents. These findings highlight the need to consider the influence of larval ecology and hydrodynamic processes to understand drivers of range‐wide connectivity.

Increasingly, studies seeking to delineate patterns of marine population connectivity are using complimentary approaches: passive particle hydrographic modelling and estimates of gene flow via next‐generation sequencing techniques, such as single nucleotide polymorphisms (SNPs) (Gormley et al., 2015; O'Dwyer et al., 2021). Modelling outputs (i.e. particle trajectories) can be compared with estimates of gene flow, allowing observed genetic patterns and expected modelled larval trajectories (Foster et al., 2012) to be considered together. Across both fine and broad‐scale distances, ocean currents have been shown to be a key driver of larval dispersal distance in many invertebrate taxa, including the common cockle (Coscia et al., 2020), sea cucumber (Xuereb et al., 2018) and many corals (Baums et al., 2006; López‐Márquez et al., 2019; Yesson et al., 2018).

This study combines population genomics and oceanographic modelling to investigate patterns of marine connectivity in *E. verrucosa*. Specifically, this study addresses the following questions: (i) what are the patterns of genetic connectivity across this species' range in the northeast Atlantic and the Atlantic–Mediterranean basin; (ii) using passive particle modelling, how do PLD and oceanography affect patterns of particle dispersal; and (iii) do patterns of passive particle dispersal agree with patterns of genetic variation in the pink sea fan?

## 2 METHODS

### 2.1 Sampling

**FIGURE 1:**
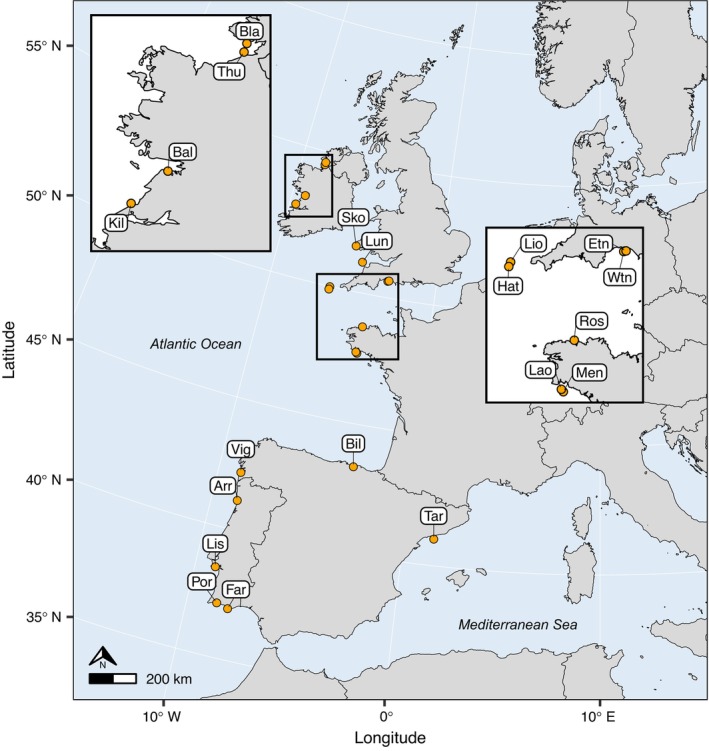
Map of the study area and sampling sites (*n* = 20) across the northeast Atlantic and Mediterranean. See Table 1 for detailed information about sites.

Tissue samples (*n* = 285) were collected via SCUBA from depths ranging between 5 and 35 m between 2007 and 2019 (Table 1). In brief, apical 5 cm cuttings were removed from each sea fan colony underwater and preserved in >95% ethanol immediately after surfacing to prevent DNA degradation. Samples were stored long term at 4°C. Tissue samples were collected from 20 sampling locations, 13 of which had been characterized previously with microsatellites (Holland et al., 2017), and seven additional sites covering much of the species' geographic range across the northeast Atlantic (Table 1). To investigate between‐basin connectivity, samples from one site (Tarragona) were collected from the Mediterranean Sea (Figure 1). As *E. verrucosa* is a legally protected species in the UK (Wildlife and Countryside Act 1981), tissue samples were collected under a sampling license granted by the Marine Management Organisation (L/2019/00143).

**TABLE 1:** Summary of sampling locations, number of colonies for *E. verrucosa* SNP study and genetic diversity metrics (*H*_obs,_ *H*_exp_ and *F*_IS_) calculated for 7510 putatively neutral SNPs.

Region	Code	*N*	LAT	LON	Year	*H*_obs_	*H*_exp_	*F*_IS_
Ireland								
Kilkee	Kil	18	52.68	−9.66	2019	0.18	0.20	0.11
Ballyvaughan	Bal	16	53.14	−9.66	2019	0.16	0.19	0.14
Thumb Rock, Sligo	Thu	13	54.47	−8.44	2012	0.16	0.21	0.16
Black Rock, Donegal	Bla	9	54.57	−8.42	2012	0.14	0.20	0.24
Britain								
Lundy Island	Lun	13	51.17	−4.68	2009	0.14	0.20	0.24
Skomer Island	Sko	14	51.74	−5.27	2007/09	0.18	0.21	0.11
East Tennants, Lyme Bay	Etn	9	50.65	−2.87	2009	0.17	0.21	0.13
West Tennants, Lyme Bay	Wtn	12	50.64	−2.96	2009	0.18	0.21	0.12
Hathor Wreck, Isles of Scilly	Hat	9	49.83	−6.33	2009	0.13	0.20	0.29
Lion Rock, Isles of Scilly	Lio	7	49.98	−6.31	2009	0.13	0.20	0.26
France								
Roscoff, north Brittany	Ros	14	48.74	−3.96	2010	0.18	0.21	0.11
Laonegued, Glénan Brittany	Lao	8	47.71	−4.06	2010	0.14	0.20	0.22
Men Goë, Glénan, Brittany	Men	14	47.69	−3.99	2010	0.15	0.21	0.21
Portugal								
Portimão, Algarve	Por	10	37.03	−8.35	2010	0.14	0.20	0.26
Faro, Algarve	Far	10	36.98	−7.99	2010	0.14	0.20	0.26
Lisbon	Lis	9	38.62	−9.24	2019	0.16	0.20	0.14
Arrábida	Arr	10	41.13	−8.67	2009	0.17	0.20	0.14
Mediterranean Spain								
Tarragona, Catalonia	Tar	22	41.08	1.22	2018	0.18	0.21	0.11
Atlantic Spain								
Vigo	Vig	26	42.19	−8.82	2019	0.18	0.20	0.13
Bilbao	Bil	3	43.36	−3.10	2019	0.16	0.20	0.01

### 2.2 DNA extraction, quality control and nextRAD sequencing

Genomic DNA was extracted from polyp tissue using a salting‐out protocol (see Jenkins, Ellis, et al., 2018) optimized for gorgonin‐protein tissue (see Appendix S1 for full protocol). The purity of all DNA extractions was assessed on a 1% agarose gel, and concentration yields were assessed using a NanoDrop 1000 spectrophotometer and via Invitrogen's dsDNA HS Assay kit and a Qubit 4 Fluorometer.

Sequencing and SNP isolation were performed through nextRAD (SNPsaurus, LLC; Russello et al., 2015), a reduced representation sequencing method. Genomic DNA was first fragmented with Nextera reagent (Illumina, Inc), which also ligates short adapter sequences to the ends of the fragments. The Nextera reaction was scaled for fragmenting 5 ng of genomic DNA, although 7.5 ng of genomic DNA was used for input to compensate for degraded DNA in the samples and to increase fragment sizes. Fragmented DNA was then amplified for 26 cycles at 73°C, with one of the primers matching the adapter and extending nine nucleotides into the genomic DNA with the selective sequence GTGTAGAGG. Thus, only fragments starting with a sequence that can be hybridized by the selective sequence of the primer will be efficiently amplified. The nextRAD libraries were sequenced on an Illumina HiSeq 4000 with one lane of 2 × 150 bp reads (University of Oregon).

### 2.3 SNP calling and filtering

Raw reads were cleaned, de‐multiplexed and trimmed to 140 bp using the process_radtags pipeline in Stacks v2.54 (Rochette & Catchen, 2017) and aligned to the pink sea fan reference genome (Macleod et al., 2023) using Bwa‐mem2 v2.2.1 (Vasimuddin et al., 2019). For all samples, the percentage of reads that aligned to the reference genome ranged from 67.6 to 98.5%. Loci were built using gstacks from Stacks with a minimum stack depth of 3 (Paris et al., 2017).

The populations pipeline of Stacks was used to retain SNPs with a minimum stack depth of 9, present in at least 16 sampling locations, minor allele frequency of 0.05 and maximum loci heterozygosity of 0.6. Further filtering using VCFtools (Danecek et al., 2011) removed genotypes containing less than 90% of SNPs, which were identified with the *missingno* function in R (R Core Team, 2023) using the package Poppr v2.8.3 (Kamvar et al., 2014), and a depth coverage <9. Loci in linkage disequilibrium were filtered in VCFtools  using a correlation (*r* ^2^) threshold of >0.7 between all marker pairs. Finally, the function *mlg* from the R package Poppr was used to identify potential clones.

### 2.4 Identifying neutral SNPs, genetic diversity and differentiation

SNPs potentially under divergent selection were detected using two outlier tests: OutFLANK (Whitlock & Lotterhos, 2015) and pcadapt v4.3.3 (Luu et al., 2017). OutFLANK estimates a distribution of population differentiation (*F*_ST_) for each SNP; loci subject to diversifying selection are expected to have higher *F*_ST_ values, whereas loci under balancing selection will have lower‐than‐average *F*_ST_ values. To minimize the False Discovery Rate (FDR), a *q*‐value of <0.05 was used. The program pcadapt is based on Principal Component Analysis (PCA) with each SNP being regressed against each principal component; SNPs are selected as outliers if they are excessively related to population structure based on *Z*‐scores. To minimize the FDR of outlier SNPs, the minor allele frequency was set to 0.01 and a 1% lower quantile value was used to select the *p‐*value cut off. To examine neutral patterns of genetic connectivity, only neutral loci common to both detection methods were retained in the dataset and considered in further analysis. For each site, observed heterozygosity (*H*_O_), expected heterozygosity (*H*_E_) and the inbreeding coefficient (*F*_IS_) were calculated using the *basic.stats* function in the R package hierfstat (Goudet, 2005). Pairwise *F*_ST_ (Weir & Cockerham, 1984) and global *F*_ST_ were calculated using the *pairwise.WCfst and wc* functions in hierfstat, respectively.

### 2.5 Population structure and gene flow

To explore population structure, three approaches were used. First, a DAPC (discriminant analysis of principal components) was performed using the *dapc* function in the adegenet package in R (Jombart et al., 2010). A DAPC summarizes genetic variation between groups, applying less weight to variation present within groups. To avoid overfitting the DAPC model, five *p*_axes_ were selected via a *k–1 criterion* (Thia, 2023) based on the number of genetic clusters identified by Holland et al. (2017) with the addition of Atlantic Spain and Mediterranean Spain, as a priori clusters.

Secondly, population structure was examined using ADMIXTURE v1.3 (Alexander et al., 2009), a model that estimates individual ancestries (*K*) based on admixture proportions (*Q*) through a maximum likelihood parametric model; *K* ranged from 2 to 21 with bootstrapping at 2000 replicates. The optimal number of ancestral (*K*) populations was selected from *K* with the lowest cross‐validation error (Alexander & Lange, 2011) and assessment of four *K* estimators: MedMeaK, MaxMeaK, MedMedK and MaxMedK, as proposed by PuechMaille (2016). Each estimator assesses the mean and median number of clusters to which at least one of the pre‐defined sampling populations belongs.

The program snapclust (Beugin et al., 2018) was also used to assess genetic clusters; this was implemented using the adegenet package in R (Jombart et al., 2010) using the default parameters. Snapclust is based on maximum‐likelihood estimations using an Expectation‐Maximization algorithm, which combines a Hardy–Weinberg equilibrium genetic model and geometric approaches (using genetic distance based on allelic data) to estimate genetic clustering (Beugin et al., 2018). The function *snapclust.choose.k* using the default information criterion (IC) was used to estimate the optimal *K* (Jombart et al., 2010).

### 2.6 Particle dispersal modelling

Oceanographic modelling was used to estimate the potential larval dispersal of *E. verrucosa* from each site sampled in the genomic analysis. *E. verrucosa* is likely to be a broadcast spawner (Munro, 2004) but the PLD and larval dispersal behaviour of this species is currently unknown.

Most studies on temperate gorgonian PLD have focused on Mediterranean brooding species and have shown that PLD is highly variable. For example, under laboratory conditions *Paramuricea clavata* larvae can drift from 8 days up to 25 days (Linares, Coma, & Zabala, 2008), while *Corallium rubrum* larvae have been shown to survive up to 42 days with a median longevity of 32 ± 11 days (Martínez‐Quintana et al., 2015). Likewise, a study simulating the larval dispersal of *P. clavata* and *Eunicella singularis* ran simulations across a 28‐day period (Sciascia et al., 2022). A review of PLD and genetic metrics found that passive dispersal of particles overestimates dispersal capacity if larval behaviour is not incorporated into the study (Selkoe & Toonen, 2011). Similar to larval behaviour, the reproductive strategy (i.e. a broadcast spawner or brooder) is not an accurate estimator of PLD (Guizien et al., 2020). Based on this information, 14‐day and 21‐day PLD simulations were chosen to estimate the passive dispersal of *E. verrucosa*, representing the central range of octocoral PLDs and limiting the overestimation of dispersal capacity from passive simulations.

Research examining the possible spawning periods of pink sea fan populations in southwest England indicated possible spawning events in line with peak sea surface temperatures (Munro, 2004). Given this, to account for potential thermal variation in spawning time across the sampling range, particles from Tarragona (Mediterranean Spain) and Portugal were released and tracked across a 21‐day dispersal window from 17th August to 7th September; for all other sites, particles were tracked from 24th August to 14th September. Simulations were run during these periods for all years from 2010 to 2020.

Passive particle tracking simulations were performed in R using an Atlantic‐Iberian Biscay Irish‐Ocean Physics analysis and a Forecast oceanographic model from CMEMS (see Appendix S1 for model details). For each sampling site, 5000 particles were released at 12:00 noon on the first day of the simulation period and left to drift in the model for 21 days in total. The simulation was run for consecutive years using oceanographic data from 2010 to 2020. To examine the potential exchange of larvae between sites, dispersal probabilities between each pair of sites were calculated to produce a connectivity matrix for a 14‐day and a 21‐day PLD. Dispersal probabilities were calculated as the mean proportion of particles that entered the source zone (20 km buffer zone; see Galindo et al., 2006) of each site; particle counts in each source zone were represented as a proportion of the particles that remained in the simulation. To represent the number of ‘larvae’ potentially self‐seeding, the proportion of particles remaining in the source zone of each site was also calculated.

### 2.7 Isolation by distance

To test for a correlation between geographic distance and genetic distance, a Mantel test was performed on pairwise *F*_ST_ and marine least‐cost distances (km) via coasts. Pairwise geographic distances between sites were calculated using the *lc.dist* function from the R package marmap v1.0.6 (Pante & Simon‐Bouhet, 2013). To investigate the presence of isolation by distance (IBD), Mantel tests were performed between dissimilarity matrices of genetic distance (*F*_ST_) and geographic distance (km) using the *mantel.rtest* function in the R package ade4 v1.7.18 (Dray & Dufour, 2007).

### 2.8 Spatial structure and environmental variables

Spatial eigenfunction analysis was performed using distance‐based Moran's eigenvector maps (dbMEMs) to assess the contribution of spatial structure to patterns of neutral genetic variation, which were then used as predictor variables in down‐stream regression analyses, following the approach used by Benestan et al. (2016). dbMEM spatial variables were calculated on marine least‐cost distances between sampling sites using the *dbmem* function in the R package adespatial (Dray et al., 2012).

Based on our understanding of *E. verrucosa* ecology, several environmental variables were selected to explore their possible relation to *E. verrucosa* neutral genetic variation, including sea water temperature, chlorophyll and current velocity at the sea surface and bottom depths (see Table S1 for data source, temporal extent and spatial resolution of each environmental variable).

To represent the possible effects of oceanic currents on genetic structure, a larval connectivity matrix of dispersal probabilities was produced based on the particle dispersal estimates between each sampling location from the particle tracking and oceanographic model. Asymmetric eigenvector maps (AEMs), a spatial eigenfunction method (Blanchet et al., 2011), were produced from a weighted site‐by‐edge matrix translated from the dispersal probabilities; 14 AEM vectors were produced reflecting the physical oceanic connectivity between the 20 sampling sites. AEM vectors were produced using the R package adespatial (Dray et al., 2012) function *adespatial::aem*.

### 2.9 Redundancy analysis: Integrating genetic structure with environment

The relative effect of selected dbMEM and environmental variables on neutral patterns of genetic variation was assessed using redundancy analysis (RDA). For the RDA response variable, allele frequencies were produced in PLINK v1.07 (Purcell et al., 2007) and Hellinger‐transformed (Borcard & Legendre, 2002) in R using the function *decostand* in the R package vegan (Oksanen et al., 2007). A PCA was performed on the transformed allele frequencies using the function *prcomp* in R package stats. Retained PC axes were selected by the Kaiser‐Guttman rule (Yeomans & Golder, 1982) and explained at least 5% of the genetic variation. RDAs and partial‐RDAs were run using the *rda* function in the R package vegan (Oksanen et al., 2014). Statistically significant explanatory variables were identified using the *ordistep* function performed with 10,000 permutations, and correlation between variables was assessed using the *vif.cca* function, from the R package vegan. To assess the significance of the global RDA model, each explanatory variable and RDA axis, an analysis of variance (ANOVA) was performed using 999 permutations.

## 3 RESULTS

### 3.1 Genotyping, quality control and filtering

After initial filtering in populations, 23,111 SNPs were identified in 285 individuals. After filtering out individuals and loci with greater than 10% missing data and monomorphic SNPs in VCFtools, 8906 SNPs in 246 individuals were retained. Additionally, 1256 SNPs were removed from each pair of SNPs identified in linkage disequilibrium. No duplicated genotypes were detected. The final dataset consisted of 7650 SNPs in 246 individuals.

### 3.2 Identifying neutral SNPs, genetic diversity and differentiation

For outlier analysis, at a significance level of *p* < 0.05, OutFLANK and pcadapt detected 7526 and 7573 SNPs as putatively neutral, respectively. Only neutral SNPs common to both outlier methods were used in further analyses, resulting in a retention of 7510 SNPs (98%) for the final data set.

*H*_obs_ ranged from 0.13 to 0.18 and was lower than *H*_exp_ in all sampled populations (Table 1). In accordance with this, estimates of *F*_IS_ were positive for all populations, ranging from 0.01 (Bil) to 0.29 (Hat) and a Global *F*_IS_ of 0.2 (Table 1), indicating a possible heterozygote deficiency within each sampling location.

Global *F*_ST_ was 0.018 and pairwise *F*_ST_ was greatest within the northeast Atlantic basin between pairs featuring Portuguese sites (Far and Por) and Irish sites (Kil, Bla and Bal) for which comparisons ranged from 0.03 to 0.033. Differentiation was generally lowest among pairwise comparisons between the British sites, with the lowest differentiation detected between Hat and Lio within the Isles of Scilly (*F*_ST_ < 0.0001). Unlike the majority of pairwise comparisons, comparisons between pairs of sites featuring Bil (Atlantic Spain), did not fully align with geographical positioning, with the lowest differentiation detected between southern Portuguese sites (Far and Por) and Bil (*F*_ST_ = 0.005 and *F*_ST_ = 0.003, respectively). Pairwise comparisons between the Mediterranean population (Tar) and the northeast Atlantic sites ranged from 0.017 (Lis) to 0.031 (Bla) (Figure S1).

### 3.3 Population structure

**FIGURE 2:**
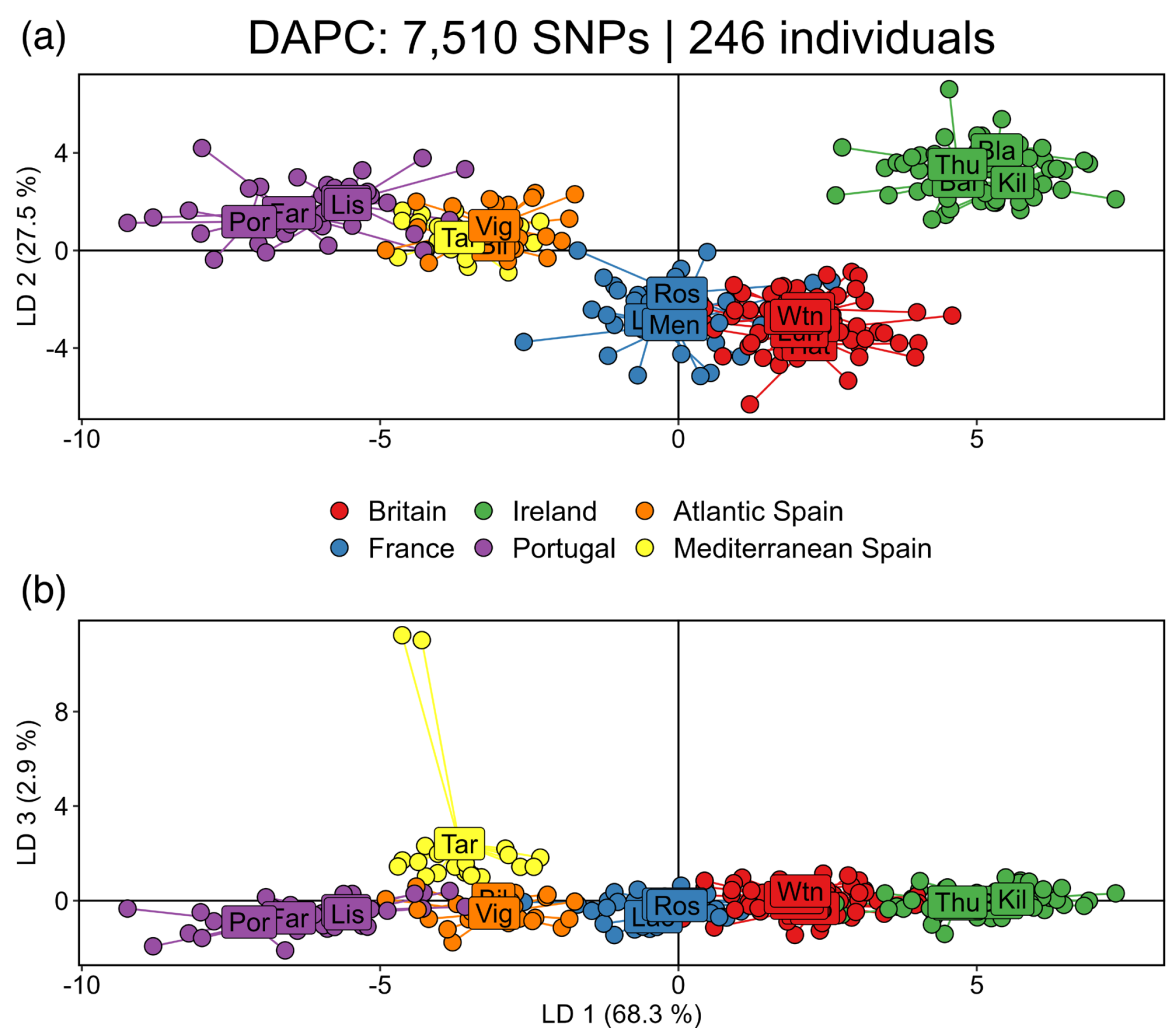
Discriminant analysis of principal components (DAPC): (a) LD 1 and LD 2, (b) LD 1 and LD 3. Each point represents an individual and colours denote from which region the individual originates.

The DAPC performed on all 7510 neutral SNPs captured 98.7% of the genetic variation across linear discriminant axis (LD) LD 1, LD 2 and LD 3 (Figure 2). Within the northeast Atlantic, LD1 (68.3%) showed support for a genetic cline with a discrete assortment of clusters partitioning into Ireland, Britain, France, Atlantic Spain and Portugal (Figure 2a). Axis LD 2 (27.5%) highlighted greater genetic structuring between Ireland and neighbouring regions, Britain and France (Figure 2a). For the Mediterranean site (Tar), individuals aligned with the Atlantic Spain sites and to a lesser extent with Lis on LD 1 and LD 2. Axis LD 3 captured 2.9% of the variation and showed slight genetic structuring between Tar and the northeast Atlantic sites, but did identify two individuals from Tar as highly differentiated from the rest of the individuals in this population (Figure 2b). In accordance with estimates of *F*_ST_, the greatest genetic variation overall was detected between individuals from Por, southern Portugal, and sites in Ireland.

**FIGURE 3:**
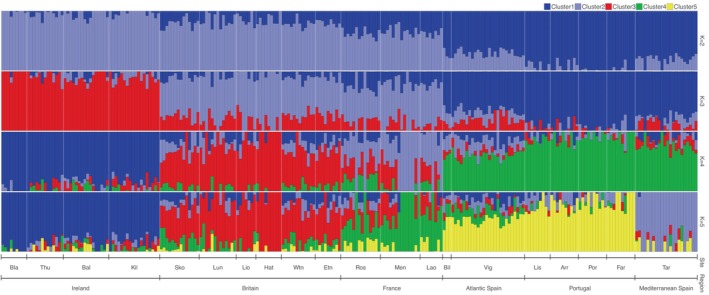
Admixture analysis for *K* = 2 to *K* = 5. Each bar represents an individual and each colour denotes which genetic cluster that individual has been assigned to. The plots show clustering patterns with no prior on location (see Appendix S1 for further details).

For clustering analysis, both ADMIXTURE and snapclust showed greatest support for *K* = 2 via the cross‐validation method (Figure S2) and AIC method (Figure S3), respectively. Selection of *K* using the Puechmaille estimators indicated an optimum of *K* = 7; as a result, values of *K* from 2 to 7 were explored visually. At *K* = 2, a clear genetic divide was detected across the sampling range with a break in admixture detected between Lao (southern Brittany, France) and Bil on the northern Iberian coast (Figure 3 and Figure S4 for snapclust). When *K* = 3 was visualized, cluster 3 grouped the Irish sites predominantly as a separate genetic cluster, differentiating this region from the rest of the northeast Atlantic sites (Figure 3). For both iterations of *K*, all genetic clusters were represented by at least one genotype across all sampling sites. With ADMIXTURE, only at *K* = 5 did the Mediterranean population, Tar, separate out as its own distinct cluster (Figure 3); the two Tar individuals shown to be highly differentiated by the DAPC were assigned solely to genetic cluster 2, which comprised predominantly Mediterranean samples. For *K* = 7, the assortment of genetic clusters generally followed the patterns at *K* = 5 but with more heterogeneous sub‐structuring within regions (Figure S5).

### 3.4 Particle dispersal modelling

**FIGURE 4:**
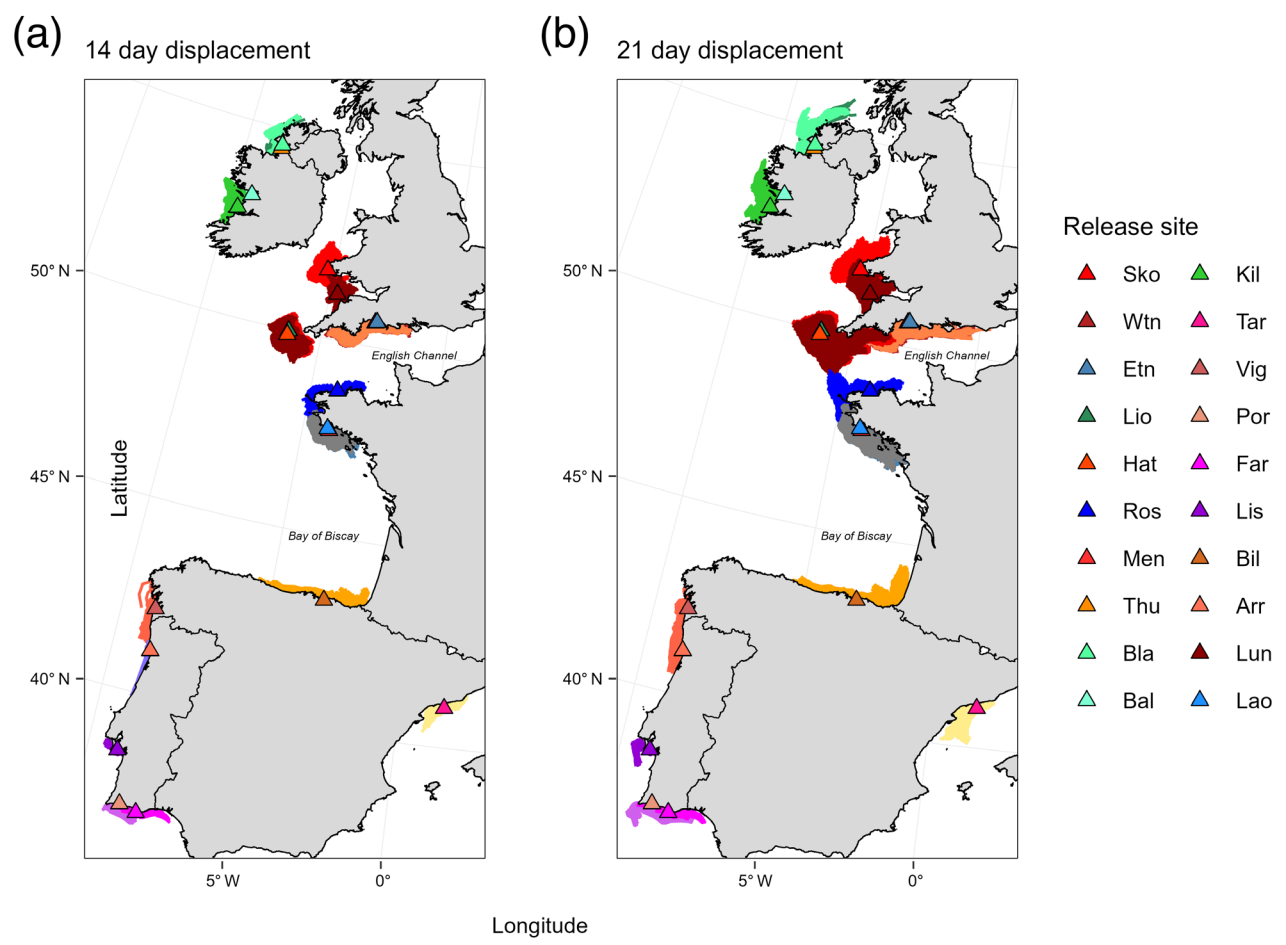
Final particle displacement trajectories for a 14‐day PLD simulation (a) and a 21‐day simulation (b). Each release site is denoted by a triangle (see corresponding key).

For both PLD scenarios (i.e. 14 and 21 days), final particle trajectory patterns indicated that the greatest potential for particle exchange generally occurred between neighbouring sites (Figure 4); however, exchange did occur between Hat (Isles of Scilly) and Ros (northern Brittany, France), under a 21‐day PLD (Figure 4b). In general, particles released from sites across southwest Britain had the greatest mean displacement distance ranging from 34.5 to 49.6 km at a 14‐day PLD and 77.2 to 103.4 km at a 21‐day PLD (Table 2). Particles released from Arr (Portugal) had the lowest dispersal for both simulations, with mean displacement distances of 5.8 km and 19.0 km, respectively.

**TABLE 2:** Passive particle dispersal statistics for each pink sea fan release site under a 14‐day and a 21‐day simulation.

Site	14‐day displacement				21‐day displacement			
	Mean number of particles remaining in simulation (%)	Mean proportion of particles remaining in release site annually (%)	Mean displacement (km)	Max displacement (km)	Mean number of particles remaining in simulation (%)	Mean proportion of particles remaining in release site annually (%)	Mean displacement (km)	Max displacement (km)
Ireland								
Kilkee	30.4	33.3	38.9	143.7	22.4	31.2	72.0	193.0
Ballyvaughan	17.5	95.8	11.5	61.2	2.1	84.3	20.0	120.2
Black rock	23.0	73.8	10.7	129.8	6.2	43.2	27.2	209.9
Thumb rock	29.3	61.8	17.7	145.5	5.2	1.8	32.7	223.8
Britain								
Lundy	91.8	40.0	34.5	104.7	79.6	10.1	77.2	186.4
Skomer	71.8	20.2	46.0	137.9	56.7	4.7	86.9	208.3
East Tennants	53.3	27.5	47.4	183.8	29.4	13.5	95.1	299.6
West Tennants	58.0	2.7	49.6	184.8	35.8	1.1	98.5	285.9
Hathor wreck	91.8	20.5	41.7	146.9	89.4	4.8	99.4	257.7
Lion rock	80.1	4.5	42.9	146.9	75.9	2.2	103.4	268.7
France								
Roscoff	22.7	0.0	29.7	179.0	12.0	0.0	63.4	328.1
Laonegued	71.1	30.6	36.7	135.9	41.4	16.1	74.0	187.4
Men Goë	74.0	0.0	37.5	144.9	44.6	0.0	75.7	218.9
Portugal								
Portimão	20.1	10.7	33.0	134.7	5.2	9.6	46.2	223.7
Lisbon	15.6	57.7	9.7	73.5	3.5	0.6	19.8	158.8
Arrábida	8.1	64.4	5.8	180.1	0.0	1.0	19.0	180.1
Faro	14.9	33.2	25.4	126.5	3.7	60.4	42.0	205.2
Mediterranean Spain								
Tarragona	60.6	34.5	23.86	96.51	43.71	9.3	56.7	163.2
Atlantic Spain								
Vigo	11.2	46.7	34.5	182.4	9.1	12.1	64.7	235.1
Bilbao	27.6	27.0	36.7	216.7	9.7	0.8	66.7	278.5

Comparison of dispersal probabilities indicated that, generally, a greater number of particles were retained within the source zone of each release site under a 14‐day PLD than a 21‐day PLD (Figure S6). Across regions, particle dispersal was lower in three of the four Irish release sites, with mean displacement distance ranging from 10.7 to 17.7 km at 14‐days and 20.0 to 32.7 km with a 21‐day PLD (Table 2). This reduced dispersal corroborated with these sites having a higher proportion of particles that remained ‘locally’ in the source buffer zones for both simulation durations.

**FIGURE 5:**
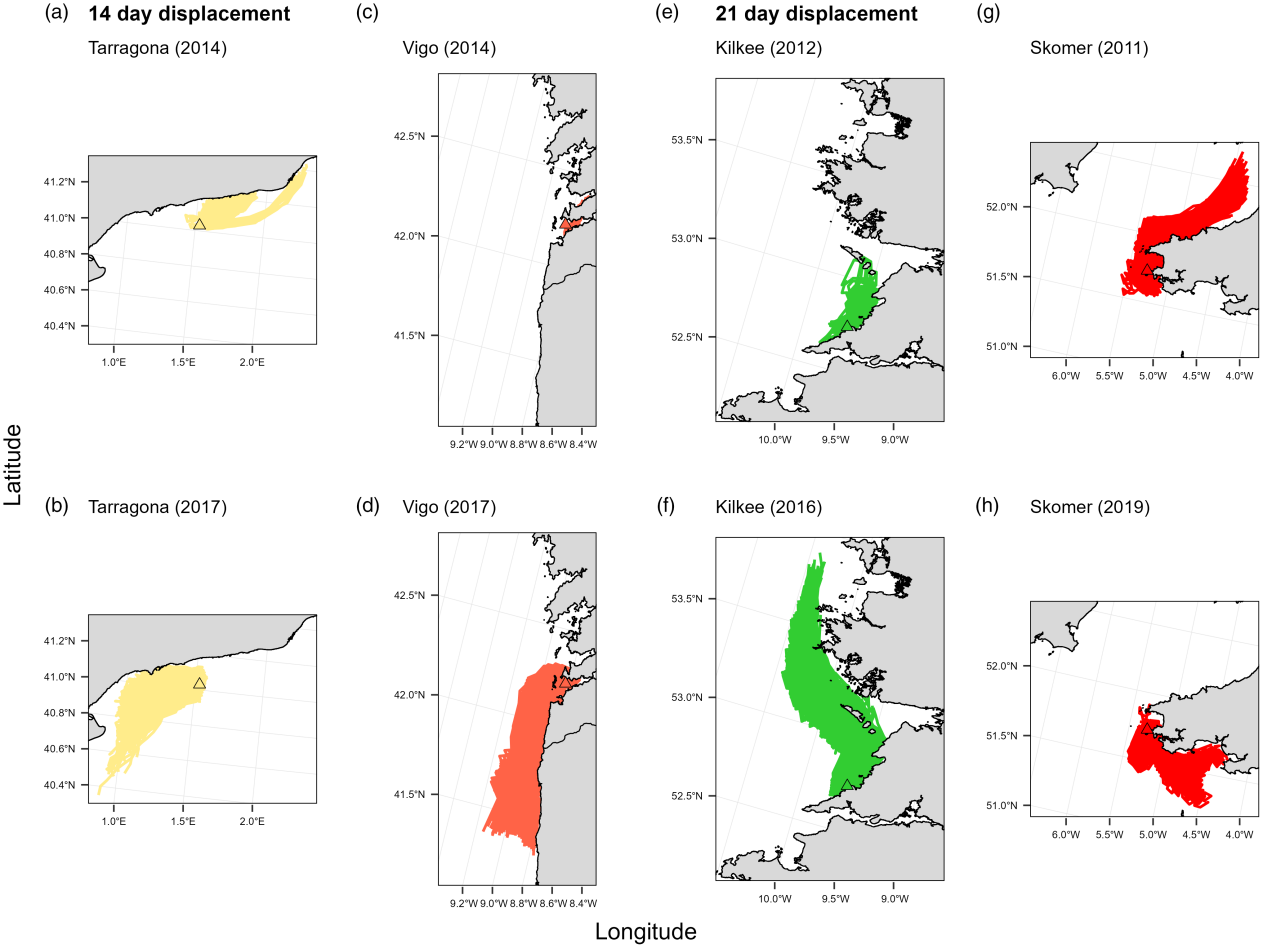
Final trajectory plots showing interannual variation in the distance and direction of dispersal trajectories for a 14‐day (a–d) and a 21‐day (e–h) drift simulation length.

For certain sites, yearly simulations highlighted significant inter‐annual variation in both the displacement distance and the direction of particles, irrespective of PLD. Maximum displacement was at least three‐fold greater and two‐fold greater than the mean displacement distance for both the 14‐day PLD and 21‐day PLD simulations, respectively (Table 2). For example, in 2014, particles released from site Vig (Atlantic Spain) with a 14‐day PLD had a max displacement distance of 23.9 km compared to a max distance of 130.9 km in 2017 (Figure 5c,d). A similar pattern of interannual variation was also found for Kil (western Ireland) (Figure 5e,f). For Thu (northwest Ireland), under a 21‐day PLD four out of the 11 simulations enabled much greater northward dispersal, with particle trajectories able to disperse a maximum distance of 223.8 km towards the Isle of Islay on the southwest coast of Scotland (Figure S7). For inter‐annual variation in trajectory direction, particles released from Tar (Mediterranean) in 2014 travelled in a northeast direction, with a max distance of 89.7 km (Figure 5a); this contrasts to the south‐westerly dispersal of particles from this site in the 2017 simulation (Figure 5b). A second example of interannual variation in particle trajectory is also shown for Sko, southwest Wales (Figures 5g,h).

**FIGURE 6:**
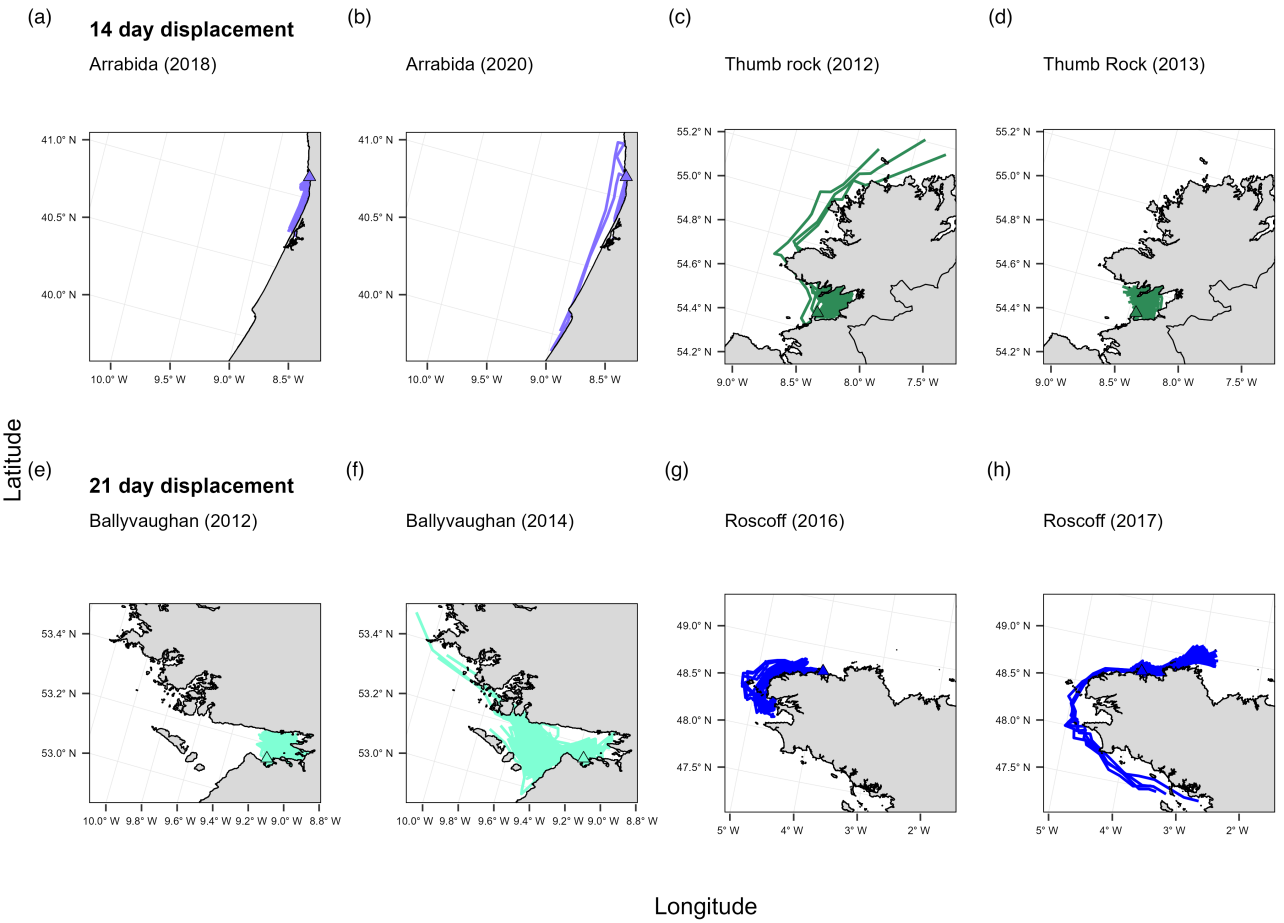
Final particle trajectory plots showing examples of infrequent, longer dispersal between years for a 14‐day (a–d) and a 21‐day (e–h) drift simulation length.

Yearly simulations also revealed a low frequency of longer dispersal events, often represented by only a single number of particle trajectories (<10), irrespective of PLD. For example, for a 14‐day PLD, oceanographic conditions in 2020 at release site Arr (Portugal) enabled a small number of particles to displace much further south (Figure 6b) compared to the average trajectory pattern of particles released in all other years, e.g. 2018 (Figure 6a). For Thu (northwest Ireland), 2013 particle trajectories represented a ‘normal’ pattern of dispersal (Figure 6d), while in 2012 a small number of particles were displaced much farther to the north around the northwest Irish coast (Figure 6c). For a 21‐day drift time, particles released in 2014 from Bal (western Ireland) were able to disperse a greater distance ‘offshore’ (Figure 6f) compared to particles modelled in 2012 (Figure 6e). Similarly, particles released at Ros (northern Brittany, France) in 2017 moved southward around Finistère towards sites in the Glénan archipelago (Men and Lao), southern Brittany (Figure 6h), compared to typical displacement patterns in other years, for example, 2016 (Figure 6g).

Finally, particles that collided with land were assumed to ‘die’ and were therefore removed from the model. As a result, across both PLD simulation lengths, for some years and sites, no particles remained in the model at the end of the simulation period (Figures S8–S10).

### 3.5 Isolation by distance

**FIGURE 7:**
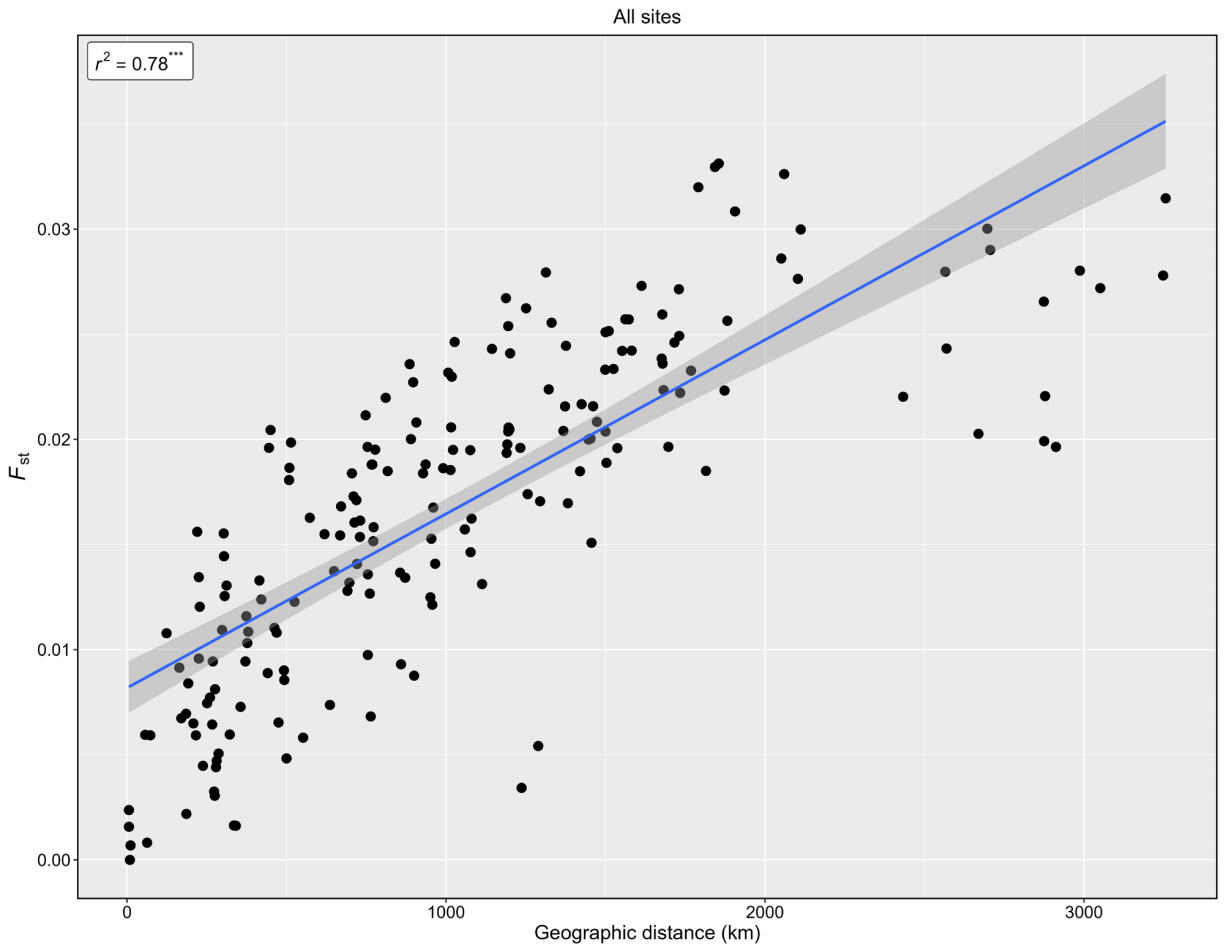
Isolation by distance (IBD) analysis of pairwise comparisons of geographic distances (km) and *F*_ST_ between all sampling sites.

A significant, positive correlation was detected between *F*_ST_ and geographic distance (*r* ^2^ = 0.78, *p* < 0.001) across individuals from the 20 sample sites, indicating isolation by distance (Figure 7). Removal of Tar (western Mediterranean) from the analysis resulted in only a slight increase in the correlation between *F*_ST_ and geographic distance (*r* ^2^ = 0.83, *p* < 0.001) among the northeast Atlantic sites (Figure S11). As the DAPC and ADMIXTURE analysis suggested increased genetic differentiation in the Irish sites, this region was also removed from the analysis, however, a clear signal of IBD remained (*r* ^2^ = 0.82, *p* < 0.001).

### 3.6 Redundancy analysis

A total of 19 dbMEM vectors were produced to represent the spatial structure between the 20 sampling locations. For the environmental variables, several variables related to water temperature showed a correlation score >0.7, resulting in the following variables being removed from the analysis: mean annual sea surface temperature, mean annual sea benthic temperature and mean benthic temperature coldest month. In addition, mean annual sea surface salinity and topography were correlated with mean annual sea bottom salinity and mean annual chlorophyll, respectively, and were also removed.

Three PC axes were retained following a PCA on the 7510 neutral SNPs, accounting for >80% of the observed genetic variation. For the RDA, the *ordistep* function selected five dbMEM vectors (MEM 1, MEM2, MEM5, MEM14, MEM 19) and two environmental variables (mean sea surface temperature coldest month and mean sea surface temperature hottest month) as significant predictors of the observed PC axes. No AEM variables, representing oceanic dispersal, were shown to be significant predictors of genetic variation. Of the selected variables, MEM1 and mean sea surface temperature coldest month were highly correlated, with a variance inflation factor >10; subsequently, mean sea surface temperature coldest month was excluded from the final RDA. The final RDA model, including spatial dbMEM predictors MEM1, MEM2, MEM5, MEM14 and MEM19 and the environmental variable mean sea surface temperature hottest month, was globally significant (*p* = 0.001) with an adjusted coefficient of determination of ($R_{\mathrm{adj}}^{2}$) of 0.87. The first two axes (RDA 1 and RDA 2) were statistically significant (*p* < 0.05) and explained 52.8% and 32.2% of the genetic variation, respectively (Figure S12). A partial RDA showed that all db‐MEM vectors and mean sea surface temperature hottest month were still significant predictors (*p* < 0.05); however, spatial db‐MEMs had much greater explanatory power and accounted for 71.3% of the neutral genetic variation, while mean sea surface temperature hottest month accounted for only 7.8% of variation (Table S2).

## 4 DISCUSSION

This study combines genome‐wide SNP markers and particle dispersal modelling to delineate patterns of population connectivity in the pink sea fan (*E. verrucosa*) and is, to date, the most comprehensive study of the species' genetic diversity across the northern part of its geographic range. Given the legally protected status of pink sea fan in some countries (e.g. the UK) and its status as a protected feature in MPA designation, characterizing patterns of population connectivity and potential drivers of such patterns are fundamentally important in directing both current and future conservation measures, for example, the designation of MPAs.

Building on the findings of Holland et al. (2017), this study extended the sample range and greatly increased sample coverage across the species' northern range. This allowed better exploration of genetic diversity and connectivity without the limitations associated with large gaps in our sample collection, which appear to have limited the conclusions of previous research. This approach has allowed us to demonstrate how genetic connectivity is characterized by range‐wide IBD from the northwest Atlantic (northwest Ireland) to the western Mediterranean (Tarragona). Particle dispersal modelling provided novel insights into the potential larval dispersal dynamics of this species and provided additional insights to interpreting the patterns of neutral genetic variation observed. Modelled displacement trajectories suggest that genetic patterns of IBD are possibly shaped by a combination of stepping‐stone connections, successive dispersal events and (infrequent) oceanographic conditions that drive rare, longer‐distance dispersal of particles.

### 4.1 Isolation by distance and connectivity

#### 4.1.1 Northeast Atlantic genetic structure

Genome‐wide analysis of pink sea fan genetic diversity provides evidence of a cline in genetic population structure across the northeast Atlantic, ranging from northwest Ireland to southern Portugal. This differs somewhat from the findings of a previous microsatellite‐based study (Holland et al., 2017) that, while recognizing the overall importance of isolation by distance, suggested other factors (including genetic drift, barriers to gene flow and selection) as potentially more dominant drivers of genetic differentiation in range‐edge populations. Correlation between geographic and genetic distance in the current study showed that genetic structure is typified by IBD (*r* ^2^ = 0.83, *p* < 0.001) to a much greater extent than suggested previously using microsatellite markers across the same geographic range (*r* ^2^ = 0.348, Holland et al., 2017). Indeed, when Irish samples were removed from the current analysis, the correlation between *F*_ST_ and geographic distance remained effectively the same (*r* ^2^ = 0.82, *p* < 0.001), suggesting that genetic differentiation detected in Irish samples using SNPs is part of a continuum of IBD across the northeast Atlantic sample sites. Spatial variables (dbMEMs), which represent the geographical positioning of sampling sites, were the greatest predictors of neutral genetic variation. For instance, MEM1, a predictor representing broad‐scale spatial patterns, described a distinct North–South cline in the northeast Atlantic sampling sites (Figure S13), which supports the hypothesis that IBD is an important driver of neutral genetic variation in the pink sea fan.

Patterns of IBD across this geographic extent have been detected in several other species, such as the netted dog whelk, with a PLD of 30–60 days (Couceiro et al., 2007), and European lobster (Jenkins et al., 2019), with a PLD of 14–28 days under laboratory conditions (Schmalenbach & Franke, 2010). The similar genetic clines observed in these other Atlantic species and knowledge of their PLDs suggest that the estimates of PLD used in our modelling studies reflect realistic estimations of *E. verrucosa* dispersal potential.

#### 4.1.2 Northeast Atlantic – Mediterranean transition

Novel insights into between‐basin gene flow suggest that genetic differentiation detected between Tarragona, western Mediterranean, and the Atlantic sites is also on a continuum of IBD, albeit with a slightly weaker but still significant correlation (*r* ^2^ = 0.78, *p* < 0.001). Thus, the Atlantic/Mediterranean transition does not appear to be driving a significant break in connectivity across this part of the range of the pink sea fan. This suggests that between‐basin genetic differentiation may be driven by the same processes (i.e. stepping stone connectivity) occurring across the broader range of *E. verrucosa* rather than an oceanic barrier to gene flow, as is the case for several other species distributed across this region (Ellis et al., 2023; Patarnello et al., 2007; Riesgo et al., 2019). This aligns with differentiation detected in the DAPC (Figure 2a), which showed the Tarragona population to cluster most closely with samples from Portuguese sites. A similar genetic pattern has been reported in *Astropecten aranciacus* (a sea star) in which genetic variation was characterized by a pattern of IBD across the Atlantic–Mediterranean boundary (Zulliger et al., 2009), while in the octocoral *Corallium rubrum* (red coral), populations sampled either side of the Almeria‐Oran front did not show any genetic break (Aurelle et al., 2011).

Patterns of ancestral clustering detected by ADMIXTURE analysis (Figure 3) were congruent with the above patterns of gene flow. The detection of common genetic clusters across all sampling regions, including between the two most distant regions (Ireland, northeast Atlantic, and Tarragona, western Mediterranean), can be explained by IBD (Figure 7) shaping gene flow in a long‐lived, low fecundity species, whereby widespread gene flow over time is facilitated through stepping‐stone dispersal. Despite this, the findings from the ADMIXTURE analysis must be interpreted with caution due to the limitations of using Bayesian clustering detection methods in the presence of marked IBD, such as over‐estimation of the number of genetic clusters present (Frantz et al., 2009; Perez et al., 2018). Similarly, the discord between the patterns obtained for Tarragona with the two clustering programs could be a result of fewer individuals sampled from the Mediterranean. However, over 90% of the genetic differentiation was captured by a DAPC in which the *p*_axes_ specification was chosen via a *K*–1 criterion (Thia, 2023) with a conservative estimate of *K* = 6.

Genetic structuring and phylogeographic patterns across the Atlantic‐Mediterranean transition are complex; an absence of a clear genetic break in pink sea fan could be explained by the Tarragona population being formed by colonization from an ancestral Atlantic population (Patarnello et al., 2007). This has been detected previously in the homogeny of assemblages of amphipods associated with biogenic reefs across the Atlantic‐Mediterranean border, suggesting some fauna in the Mediterranean Sea are of Atlantic origin (Plicanti et al., 2017). However, two individuals from Tarragona appear to not fit this trend, with both the DAPC (Figure 2b) and clustering analysis (Figure 3; *K* = 2) showing marked genetic differentiation in two colonies relative to other samples from this site. The Tarragona site is somewhat atypical: sea fans grow at relatively high density along the man‐made breakwater of Tarragona industrial harbour, a structure built and modified from the 1950s onwards. Thus, colonization of this structure may have occurred relatively recently from a number of diverse source populations (predominantly the Atlantic and areas of the Mediterranean populated with Atlantic‐lineage colonies, together with –apparently – some otherwise unsampled Mediterranean sources). As a result, only a low level of genetic differentiation may have been able to establish between the Tarragona population and Atlantic‐lineage populations over contemporary timescales. In contrast, much finer levels of genetic structuring have been found throughout the Mediterranean in other octocorals (*P. clavata*, Mokhtar‐Jamaï et al., 2011; *E. cavolini*, Aurelle et al., 2018). Clearly, further research is required to fully assess patterns of genetic variation in *E. verrucosa* across the Mediterranean Sea.

#### 4.1.3 Stepping‐stone patterns of dispersal and the importance of inter‐annual variation in long‐distance connectivity

The complimentary use of gene flow estimates and passive particle dispersal modelling in the current study has provided novel insights into understanding how patterns of stepping‐stone connectivity are achieved in a sessile gorgonian with an unknown PLD. Simulations for a 14‐day and a 21‐day PLD both suggest that dispersal occurs predominantly between neighbouring sites. Despite this, the considerable inter‐annual variation of particle direction and distance for both the 14‐day and 21‐day PLD simulations indicates an important role of local oceanography in driving longer, rarer dispersal events that are sufficient for enabling stepping‐stone connectivity over larger geographic distances. Global Lagrangian dispersal modelling of a generic broadcast spawning coral has shown that under the correct oceanic conditions, rarer between‐basin regional connectivity is maintained by extreme, long‐distance dispersal events (Wood et al., 2014), a pattern found in genetic studies of various tropical coral species (Gylnn & Ault, 2000; van Oppen et al., 2011). Similarly, in another temperate octocoral, *P. clavata*, the sharing of haplotypes and incorrect cluster assignment of individuals throughout the Mediterranean Sea were attributed to sporadic long‐distance migration events (Mokhtar‐Jamaï et al., 2011).

### 4.2 Beyond IBD: Exploring potential breaks in connectivity

#### 4.2.1 The Bay of Biscay break

Clustering analysis of the SNP data demonstrated greatest support for two ancestral genetic clusters and evidenced a genetic break between pink sea fan populations in the north of the species' range, as far south as southwest Brittany (Laonegued, Glénan Archipelago), and populations to the south around the Iberian Peninsula from Bilbao in the southern Bay of Biscay to Tarragona in the western Mediterranean. In contrast, the potential north–south genetic break detected across the Bay of Biscay was not reflected directly in the modelled particle trajectories. For a 21‐day drift time, modelled particles released from Bilbao were able to disperse over 250 km northwards across the Bay of Biscay, suggesting the potential for larvae to reach areas of northwest France and gene flow to be maintained via stepping‐stone connectivity.

However, the break detected in the genetic data may be due to hydrographic features across this expanse. The topography and hydrodynamic processes across the Bay of Biscay differ greatly in the northern and southern regions and have been described as two separate sectors (Koutsikopoulos & Le Cann, 1996). The Bay of Biscay is hydrodynamically complex due to the influences of river plumes, salinity variation, upwelling dynamics and wind patterns (Koutsikopoulos & Le Cann, 1996), potentially enabling dispersal only under certain hydrographic conditions (Ayata et al., 2010; Kelly‐Gerreyn et al., 2006). Population structuring suggestive of breaks in connectivity has also been detected in several other species across this region: a seaweed, *Fucus cerenoides* (Nicastro et al., 2020) and several polychaete species (Ayata et al., 2011). Indeed, the break in gene flow observed in our SNP data mirrors a reported geographical break in the distribution of *E. verrucosa* populations south of the Glénan Archipelago (obis.org), although single sightings have been recorded as far south as La Rochelle (iNaturalist, 2023). This pattern is possibly driven by a lack of suitable substrate for pink sea fan across the Bay of Biscay which is characterized by sand and muddy sand habitat (ICES.dk), especially in the southeast coastal region of the bay. No known sightings of pink sea fan have been recorded in this region at SCUBA‐accessible depths; however, it cannot be ruled out that populations may be present at deeper depths.

#### 4.2.2 Connectivity across the English Channel

Estimates of particle dispersal using a 21‐day simulation suggested connectivity between populations from Roscoff and the Isles of Scilly, showing the potential for pink sea fan larvae to disperse across the English Channel. This dispersal capacity accords with the relatively low genetic differentiation detected between these areas and is again suggestive of stepping‐stone connectivity. Similarly, dispersal simulations for a species of hermit crab, *Clibanarius erythropus*, suggested that larval transport from Brittany, northwest France, to southwest Britain is rare but possible and results from unusual ocean currents, though the authors found the Channel may still act as a barrier to species with a larval PLD of less than 20 days (Patterson et al., 2022). Similarly, Ayata et al. (2010) simulated particle dispersal trajectories with a 4‐week PLD and showed successful dispersal across the Channel due to residual circulation under realistic hydrodynamic conditions. The low genetic differentiation between these regions in the current study accords with a longer PLD (>20 days) for pink sea fan.

#### 4.2.3 Habitat suitability

Patterns of particle dispersal also highlighted areas of the species' range where factors other than dispersal capacity, for example, habitat suitability, may be acting to limit the distribution of the species. For both modelled PLDs, model trajectories indicated the potential for dispersal past the current known range of pink sea fans around southwest Britain and northwest Ireland (Holland & Stevens, 2014; Pikesley et al., 2016), in particular, the movement of particles released from Lyme Bay, southern England, into the eastern part of the English Channel and the northward dispersal of particles from Skomer Island, southwest Wales (Figure 4). Such patterns highlight the potential for other factors, such as availability of suitable physical habitat (e.g. a hard substrate) or localized environmental conditions, in shaping the distribution of *E. verrucosa*. Indeed, while sea surface temperature for the hottest month only explained 7.8% of the neutral genetic variation, the latitudinal spatial structuring among sampling sites (represented by MEM1) aligned with a cline in sea surface temperature across the study area, indicating the potential importance of temperature in driving patterns of non‐neutral structure and population connectivity. Indeed, temperature has been found to be one of the important predictors of habitat suitability for this species, alongside slope, wave orbital velocity and oxygen concentration (Jenkins & Stevens, 2022).

### 4.3 Particle dispersal modelling: Limitations

The modelled particle trajectories at each sampling site may not be an accurate reflection of pink sea fan larval dispersal potential as biological and behavioural parameters could not be incorporated into the model due to limited knowledge of this species' reproductive ecology. For some sites, a high proportion of particles were removed from the model following collision with land, thereby decreasing the modelled connectivity of these sites – such patterns may change with the integration of larval traits into the model, for example, negative buoyancy or active swimming. Despite this, a recent study by Sciascia et al. (2022) found that for gorgonians, the spatial resolution of the model had the strongest effect on larval transport, rather than the integration of larval behaviour; similar findings were also evident when examining deep‐sea connectivity (Lavelle et al., 2010; McGillicuddy et al., 2010). Given the major unknowns regarding the spawning period of *E. verrucosa*, modelled particles were released during a single event rather than across a spawning period so as to limit the number of modelled uncertainties, which may not accurately reflect the real frequency of gamete release. In addition, the model comprised oceanographic data for only one depth (~0.4 m) and, therefore, may not be a true representation of the depth at which larvae are typically present.

While the spatial scale of genetic connectivity detected in this study is much greater than the oceanographic connectivity estimated by the passive dispersal modelling, a recent review of dispersal models (Legrand et al., 2022) found that almost 70% of coalescent genetic connectivity is best predicted through a multi‐generational model of dispersal that accounts for successive dispersal events by previous generations. Such a finding accords with the lack of significant asymmetric eigen‐vector maps (AEMs) explaining neutral population structure in the current study, as AEM vectors only represent oceanic dispersal events occurring in one generation under experimental PLD scenarios and, therefore, do not capture successive ‘stepping‐stone’ dispersal events. Reduced modelled connectivity may also result from a lack of representation of all known populations of a species, or accounting for population abundance and reproductive effort; this could lead to a disjunct between genetic and dispersal‐based estimates of connectivity. However, given the known distribution of this species and its habitat preferences (Jenkins & Stevens, 2022), this is unlikely to be the case in the current study. As a result, clear signals of IBD and the sharing of genetic clusters across the species' range suggest that sea fan larval dispersal does accord with an oceanographic stepping‐stone model.

### 4.4 Implications for conservation

This study presents evidence that range‐wide population connectivity and exchange of genetic variants occurs in a stepping‐stone model, potentially underpinned by infrequent longer dispersal events shaped by oceanography. While these long‐distance dispersal events appear to support long‐term ecological connectivity between distant populations across the range of the pink sea fan, genetic diversity at a local population level appears to be less well maintained.

#### 4.4.1 Range‐wide inbreeding

The use of several thousand neutral SNPs has revealed the presence of inbreeding and low heterozygosity across all populations sampled in this study. A previous genetic study of pink sea fan in the northeast Atlantic using 13 microsatellite loci (Holland et al., 2017) reported a much patchier occurrence of inbreeding, with only 9 out of 27 populations studied showing evidence of significant inbreeding. However, low observed heterozygosity estimates can be an artefact of deriving genotypes from SNP markers and have been shown to be impacted by sample sizes and filtering thresholds for missing data at SNP loci (Schmidt et al., 2021). While such artefacts cannot be ruled out here, the possible effects of these were mitigated by filtering out those genotypes with greater than 10% missing data and retaining only SNPs with high‐quality genotypes occurring in at least 80% of sampling locations. Accordingly, we suggest that the uniform pattern of low heterozygosity detected with a genome‐wide panel of SNPs (Macleod et al., 2023) in the current study better reflects pink sea fan reproductive ecology than the very patchy range of results obtained previously with microsatellite loci (Holland et al., 2017).

Heterozygote deficiencies can also result from the presence of subdivision within a population in which allele frequencies differ spatially depending on the level of gene flow or influence of local random processes – the Wahlund effect (Garnier‐Géré & Chikhi, 2013). While we cannot rule out such a process in driving the excess homozygote genotypes detected in this study, inbreeding in marine invertebrates is suggested to be a much more common aspect of their biology than previously assumed (Olsen et al., 2020). Indeed, despite very distant evolutionary histories, strikingly similar distributions of positive *F*_IS_ values have been shown for sessile marine invertebrates, macroalgae and terrestrial seed plants, reflecting common life history traits, such as dispersal via the release of gametes into the environment, with the level of inbreeding directly related to the ability of such organisms to disperse and/or self‐fertilize (Olsen et al., 2020). Such reports highlight a greater likelihood of inbreeding as the main mechanism of non‐random mating, over the Wahlund effect, acting within the pink sea fan populations studied here.

From the methods employed in this study, it is not possible to ascertain whether this possible inbreeding across the *E. verrucosa* populations studied is a recent phenomenon or a long‐standing characteristic of this species. However, from knowledge of this species' life history (i.e. slow growth, low fecundity) and long‐term, range‐wide population persistence, we suggest this is not a recent phenomenon and is, therefore, unlikely to reflect a contemporary reduction in genetic connectivity. While this would suggest no immediate conservation concern, any significant reduction in connectivity that promotes widespread inbreeding may result in disproportionate outcomes/effects across populations with regard to the level of standing genetic variation and its importance in underpinning population responses to environmental change (Barrett & Schluter, 2008).

The effects of inbreeding, for example, genetic drift, are typically greater in smaller, isolated populations more governed by neutral processes (Kimura & Ohto, 1971; Pardo et al., 2005). This is of particular concern for the most northerly populations in this study, that is, those in northwest Ireland, whereby the effects of lower genetic diversity could be exacerbated by the apparent lower dispersal capacity, as identified by the overall shorter dispersal patterns modelled across this region. Despite this, however, rarer particle trajectories indicated the potential for these Irish populations to be a source of migrants to the western Scottish coast, an area currently devoid of pink sea fans but where suitable habitat for *E. verrucosa* has been identified (Jenkins & Stevens, 2022). Such dispersal events may underpin a northward shift of the current range‐edge as suitable habitat becomes available under a scenario of warming seas (Jenkins & Stevens, 2022). Critically, from a conservation perspective, the potential for an expanding northern range‐edge is no cause for complacency, as loss of genetic diversity through inbreeding (or any other causes) progressively limits the capacity of an organism to respond to environmental changes and associated selective pressures.

In contrast, for populations at the current southern edge of the species' range inbreeding could pose more serious problems. Typically range‐edge populations occupy areas where a species is approaching its ecological limits (Bridle & Vines, 2007). As a result, colonies at more southerly sites are likely to already be at or near their thermal maxima and the capacity to shift to more northerly, cooler water may be reduced. This is particularly pertinent for the Tarragona population in the northwest Mediterranean, as a significant range‐shift in latitude would not be possible in this region; accordingly, such populations will be restricted to exploiting deeper, cooler waters to mitigate the effects of elevated near‐surface sea temperatures. Moreover, the lack of incoming migrants (as is assumed to be the case where inbreeding is frequent) and the subsequent lack of genetic exchange could limit the adaptive potential of such populations. As noted, however, the potential for this species to survive in elevated seawater temperatures is currently unknown and further research is needed.

What does this mean for conservationists managing these range‐edge populations of *E. verrucosa*? Greater protection of leading‐edge populations in northwest Ireland – represented here by Thumb Rock (Thu) and Black Rock (Bla) in Donegal Bay through the designation of an MPA, could limit physical disturbance to these colonies and maximize the potential for a possible northerly range‐shift in this species from these donor areas. This would feed into the Irish Government's aim to have an MPA network covering 30% of its maritime area by 2030 and aligns with the EU Biodiversity Strategy for 2030 and the EU Marine Strategy Framework Directive (Government of Ireland, 2022). For trailing‐edge populations – represented here by Portimão (Por) and Faro (Far) in southern Portugal – responses to oceanic warming may rely on the ability of these colonies to adapt, either through the selection of adaptive variants, if present, or through phenotypic plasticity. While the designation of an MPA would not directly facilitate such processes, it could offer long‐term protection to these populations, safeguarding their diversity and, in turn, their capacity to adapt to change.

#### 4.4.2 Is the UK MPA network fit for the purpose of protecting pink sea fan?

Previous research into local patterns of pink sea fan gene flow supports the premise that MPA designations across southern England and Wales constitute a network for the species, suggesting they are adequately spaced to maintain and facilitate stepping‐stone genetic connectivity across this part of the species' range (Holland et al., 2017; Jenkins & Stevens, 2018). For example, mean dispersal distance from Lyme Bay sites ranged from 47.3 to 49.6 km and from 41.7 to 42.9 km in the Isles of Scilly for a 14‐day PLD. These modelled trajectories support the use of a 40 km buffer between MPA boundaries in England as deemed sufficient to maintain ecological connectivity (Roberts et al., 2010).

#### 4.4.3 Wider pink sea fan range

The results of this study suggest that stepping‐stone dispersal is a likely mechanism for maintaining genetic connectivity across the broader northeast Atlantic range of this species. Although pink sea fan is currently only a specific designation feature for UK MPAs, it may be adequately protected via the EU Habitats Directive Annex 1 (habitats) across coastal regions of Ireland, France, Portugal and Spain, where present. However, estimates of gene flow in this study suggest that there may be a break in stepping‐stone connectivity between populations situated off the southern coast of Brittany, France and populations along the southern Bay of Biscay shelf. This has important policy implications for conservation bodies governing this region as it represents a potential lack of ecological coherence in the MPA network due to a natural break in suitable habitat for this species. It also highlights the need to ensure populations on the northern Bay of Biscay shelf (around Brittany and La Rochelle) receive adequate protection to mitigate their potential natural in‐range isolation and limited supply of larval recruits from the south of the range.

More broadly, this study demonstrates the importance of annual variation in oceanographic dispersal patterns in shaping genetic connectivity across pink sea fan populations. This has direct implications for the management of such populations where localized oceanography can enable rare long‐distance dispersal events (Figure 6) that are likely critical for maintaining widespread gene flow. Accordingly, management should seek to limit (and monitor) direct disturbance practices, for example, scallop dredging, and to implement No‐Take Zones that would ban activities potentially harmful to pink sea fan at these critical stepping‐stone sites. In addition, genetic connectivity in the pink sea fan may be limited in areas that represent natural breaks in habitat for the species, highlighting the need to consider such ecological information in any future designation of MPAs concerning this species, and across other marine invertebrates more generally.

## CONFLICT OF INTEREST STATEMENT

None declared.

## Supporting information


Appendix S1

## Data Availability

Underlying RADseq reads are available at the European Nucleotide Archive (ENA) under the study accession PRJEB71722. Filtered data sets and R scripts for the analyses are available from the Dryad Digital Repository: https://doi.org/10.5061/dryad.xwdbrv1m9.
