# Supplementary material for: Rare, long‐distance dispersal underpins genetic connectivity in the pink sea fan, Eunicella verrucosa
Source: Evol Appl. 2024 Mar 7;17(3):e13649. doi: 10.1111/eva.13649 (PMC10918604; doi:10.1111/eva.13649)
Supplement: Supplementary file 1 — Appendix S1 [file EVA-17-e13649-s001.docx]

**Appendix S1: Supplementary information**

**Methods**

**Population structure**

The optimal number of ancestral (*K*) populations was selected from *K* with the lowest cross-validation error (Alexander and Lange, 2011) and assessment of four *K* estimators: MedMeaK, MaxMeaK, MedMedKand MaxMedK proposed by Puechmaille (2016); each estimator assesses the mean and median number of clusters to which at least one of the pre-defined sampling populations belongs to. This method has been shown to account for potential uneven sample sizes; this was considered important due to nine populations in this study having fewer than 10 individuals.

**Particle dispersal modelling**

Passive particle tracking simulations were performed in R using an Atlantic-Iberian Biscay Irish-Ocean Physics analysis and a Forecast oceanographic model from CMEMS (see Supplementary Material for model details) (product ID: global-analysis-forecast-phy-001-024-hourly-t-u-v-ssh). The model variables represented the eastward and northward current velocity at a depth of ~0.4 m at a spatial resolution of 0.083° × 0.083° with a daily temporal resolution. The hourly time step for each particle was converted into a shapefile; particles which overlapped with land were removed from the model. Particle trajectories were extracted from each shapefile and visualised.

**Spatial structure and environmental variables**

To characterise the spatial structure of *E. verrucosa* across the study area, and to assess the contribution of spatial structure to patterns of neutral genetic variation, spatial eigenfunction analysis was performed using distance-based Moran’s eigenvector maps (dbMEMs). Firstly, in-water least-cost geographic distances between sampling sites were obtained using the *lc.dist* function in R package marmap [(Pante & Simon-Bouhet, 2013)](https://www.zotero.org/google-docs/?ZSxbfi) and were converted into a distance matrix using the *dist* function in R package stats (R Core Team, 2020). dbMEM spatial variables were calculated on the matrix of marine least-cost distances using the *dbmem* function in R package adespatial [(Dray et al., 2012)](https://www.zotero.org/google-docs/?dGdL3W) and summarise the spatial structure from a distance matrix across scales, representing the spatial relationships among all sampling sites [(Borcard & Legendre, 2002)](https://www.zotero.org/google-docs/?4jz0oZ). Each dbMEM vector can then be used as a predictor variable and control for spatial correlation when testing for genetic-environmental associations in down-stream regression analyses [(Benestan et al., 2016)](https://www.zotero.org/google-docs/?i1cW3O).

The selection of environmental variables to explore in relation to neutral genetic variation were guided by our current understanding of pink sea fan ecology and distribution, and the reproductive ecology in corals more widely. Sea water temperature is a key ecological variable linked to the initiation of spawning in tropical coral species and can influence the development dynamics and settlement cues of the dispersing larval stage for a suite of species [(Bruge et al., 2016; Keshavmurthy et al., 2014)](https://www.zotero.org/google-docs/?oMlVTZ). In addition, findings from Holland et al*.* (2017) indicate the potential for range-edge selection pressures, such as more extreme minimum and maximum temperatures, in driving connectivity in *E. verrucosa.* To represent annual variation in sea water temperature, in particular the minimum and maximum temperatures across the species’ distribution, mean temperature for the coldest month (March) and hottest month (August) for both sea surface and bottom temperature were estimated and used as environmental variables downstream. Additional variables thought to be relevant to *E. verrucosa* larval ecology included: sea surface current velocity (SCV), sea surface salinity (SSS), sea bottom current velocity (BCV), sea bottom salinity (SBS), chlorophyll concentration (CHL) and topography. For all non-static variables, a mean of monthly data was used. The data source, temporal extent and spatial resolution of each environmental variable are listed in **Table S1**. Potential correlations between the selected environmental variables were assessed using a Pearson correlation via the *cor* function in R package stats; variables shown to have a covariance greater than 0.7 were removed from the downstream analysis.

To represent the possible effects of oceanic currents on the genetic structure of *E. verrucosa,* a larval connectivity matrix was produced based on the particle dispersal estimates between each sampling location from the particle tracking and oceanographic model (see above). The particle dispersal probability was quantified as the average proportion of larvae that moved from a source population (i.e. one grid cell) to within a 20 km buffer zone of a recipient population. The total number of particles reaching each recipient population were represented as the proportion of particles left in the simulation that did not beach. Asymmetric eigen-vector maps (AEMs) are a spatial eigenfunction method developed to model multivariate spatial distributions (i.e. SNP allele frequency) generated by an asymmetric, physical process (i.e. oceanic dispersal of larvae) (Blanchet et al*.*, 2011). AEM vectors were produced from a weighted site-by-edge matrix translated from the dispersal probabilities between sites within the larval connectivity matrix. A total of fourteen AEM vectors were produced reflecting the physical oceanic connectivity between the 20 sampling locations in this study. Both AEM and dbMEM vectors were produced using the R package adespatial (Dray *et al.* 2012) functions adespatial::aem and adespatial::dbmem, respectively.

**Results**

**Data filtering**

Six samples from Bilbao and 12 samples from Tarragona were removed during filtering due missing data greater than 20%; on average lower reads and on average a lower number of reads aligned to the reference genome, when compared with samples from the same location. Based on initial PCA clustering analysis and greater missing data than retained samples, a further Bilbao sample was removed from the dataset to ensure downstream analyses was not biased by these samples.

**Population structure**

For ADMIXTURE, cross validation at *K* = 2 was indicated with (CV error = 0.4729) and without (CV error = 0.4734) using sampling location as a prior.

For ADMIXTURE, at *K* = 7, individuals from the two most northerly Irish sampling sites (Black Rock and Thumb Rock, both in Donegal Bay) did appear to be well differentiated from Kilkee and Ballyvaughan, the two more southerly western Irish sites (**Figure S5**).

**Passive dispersal modelling**

Overall, oceanographic modelling for both simulation scenarios revealed that particles released from southwest Britain had the greatest dispersal capacity and could act as an important mid-range source of larvae to other populations. This is possibly due to the location of these release site and the major oceanographic influence from the English Channel and Celtic Sea. Such trajectories support the estimates of contemporary gene flow detected using microsatellite genotypes in Holland *et al.* (2017), which indicated southwest Britain sites to be a major source of alleles to all other regions.

Estimates of particle dispersal using a 21-day simulation suggested connectivity between populations from Roscoff and the Isles of Scilly sites, showing the potential for pink sea fan larvae to disperse across the English Channel. This dispersal capacity aligns with the low genetic differentiation detected between these regions and movement of larvae across this expanse would support stepping-stone connectivity in this species. Dispersal simulations for a hermit crab species, *Clibanarius erythropus*, suggested that larval transport from the Brittany region of northwest France to southwest Britain is rare but possible and results from unusual ocean currents (Patterson et al., 2022). Particle dispersal trajectories simulated for a 4-week PLD under realistic hydrodynamic conditions also showed successful dispersal across this region due to the English Channel residual circulation (Ayata et al., 2010).

Finally, particles that collided with land were assumed to ‘die’ and were therefore removed from the model. As a result, across both PLD simulation lengths, for some years and sites, no particles remained in the model (**see Figures S8-10**); for example, only in simulation years 2010, 2011, 2013, 2015 and 2019 did particles remain in simulation from release site Portimão, Portugal (**Figure S10**). Similarly, in the 21-day simulation, no particles released from Arrabida, Portugal were retained in the model after all particles ‘collided with land’.

**References (additional to main paper)**

Borcard, D., & Legendre, P. (2002) All-scale spatial analysis of ecological data by means of principal coordinates of neighbour matrices. *Ecological Modelling*, 153 (Issues 1–2): 51-68. <https://doi.org/10.1016/S0304-3800(01)00501-4>

Bruge A., Alvarez P., Fontán A., Cotano U., & Chust G. (2016) Thermal niche tracking and future distribution of Atlantic mackerel spawning in response to ocean warming. *Frontiers in Marine Science*, 3: 86. doi=10.3389/fmars.2016.00086

Keshavmurthy, S., Fontana, S., Mezaki, T. et al. (2014) Doors are closing on early development in corals facing climate change. *Scientific Reports*, 4: 5633. <https://doi.org/10.1038/srep05633>

**Spatial structure and environmental variables**

**Table S1.** Environmental variables used to characterise each sampling site for seascape analysis. All data were obtained from Copernicus.

| **Data type** | **Source** | **Spatial resolution** | **Temporal resolution** | **Temporal extent** | **Estimated environmental variable** |
| --- | --- | --- | --- | --- | --- |
| **Sea surface temperature** | Copernicus Marine Environment Monitoring System (CMEMS) | 0.05° | Daily | 1990-2016 | Mean surface temperature (SST_OM)  Mean hottest month (SST_hM)  Mean coldest month (SST_cM) |
| **Sea surface salinity** | CMEMS | 0.083° | Monthly | 1993-2013 | Mean surface salinity (SSS) |
| **Sea bottom salinity** | CMEMS | 0.083° | Monthly | 1993-2013 | Mean bottom salinity (SBS) |
| **Sea surface current velocity** | CMEMS | 0.083° | Monthly | 1993-2013 | Mean surface current velocity (SCV) |
| **Sea bottom temperature** | Bio-oracle | 0.092° | Monthly average | 2000-2014 | Mean benthic temperature (MBT)  Mean hottest month (MBT_hM)  Mean coldest month (MBT_cM) |
| **Sea bottom salinity** | Bio-oracle | 0.092° | Monthly average | 2000-2014 | Mean bottom salinity (SBS) |
| **Sea bottom current velocity** | Bio-oracle | 0.092° | Monthly average | 2000-2014 | Mean bottom current velocity (BCV) |
| **Chlorophyll concentration** | CMEMS | 4 km | Monthly | 1997-2019 | Mean Chlorophyll (CHL) |
| **Topography** | Global Multi-resolution Topography Data Synthesis | 1 km | NA | NA | Topography (TPG) |

**Table S2.** Redundancy Analysis (RDA) results for the environmental, spatial and full RDA in relation to the explanatory models selected for each model, respective

*P* values and adjusted coefficient of determination (*R*_adj_^2^) values.

| Analyses | Selected variables  (ordistep function) | | *P* model | *R*_adj_^2^ |
| --- | --- | --- | --- | --- |
|  | Environmental | Spatial |  |  |
| Full RDA | SST_hM*** |  | 0.001 | 0.37 |
|  |  | MEM1***  MEM2***  MEM5***  MEM14**  MEM19*** | 0.001 | 0.87 |
| Partial RDA (condition: SST_hM)  Partial RDA (Condition: dbMEMs) | SST_hM** | MEM1***  MEM2***  MEM5***  MEM14***  MEM19*** | 0.001  0.02 | 0.71  0.07 |

Significant explanatory variables are indicated with the following symbols.

**P* < 0.05

***P* < 0.01

****P* = 0.001

**
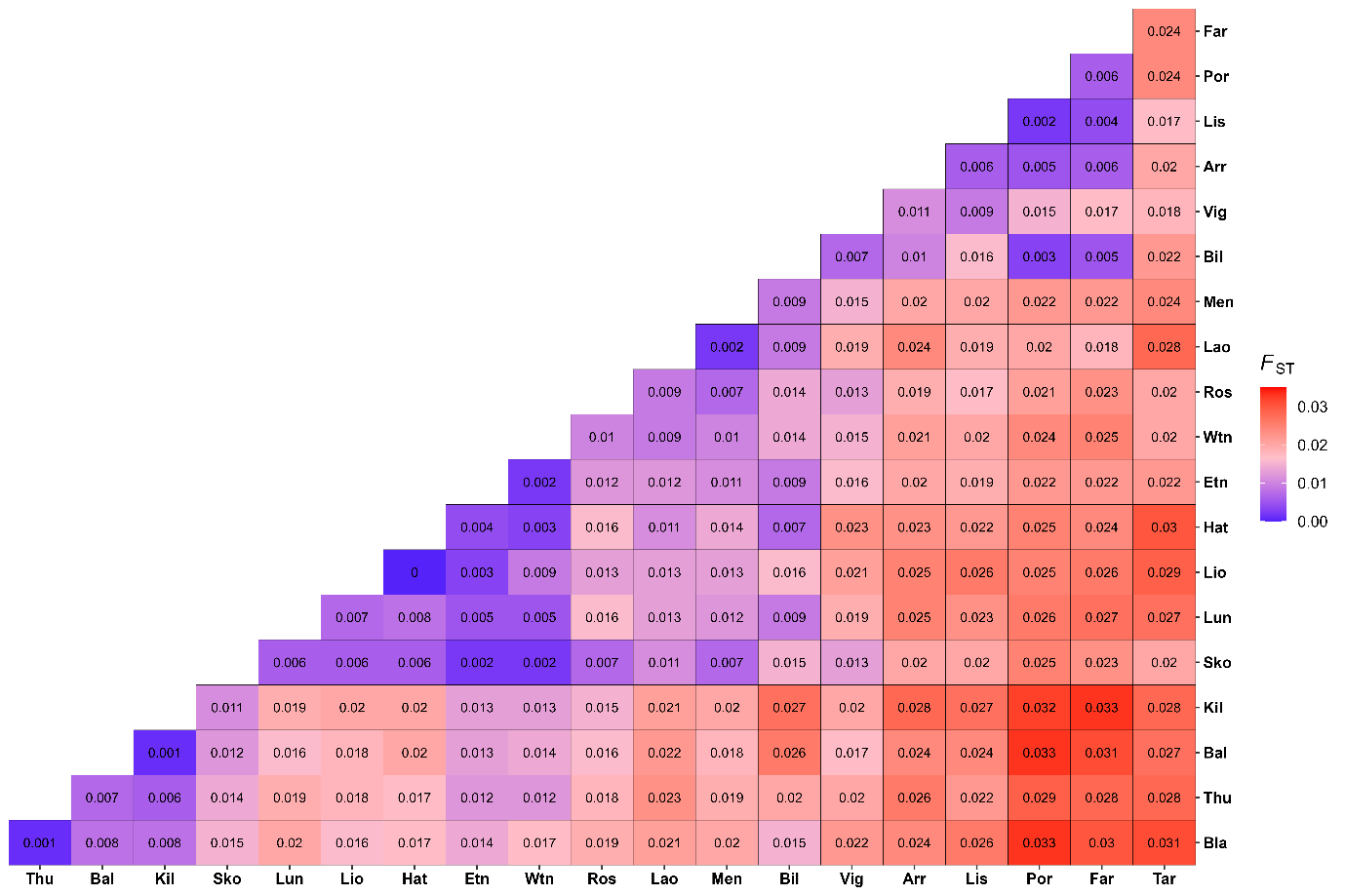
**

**Figure S1.** Weir and Cockerham *F*_ST_ pairwise comparisons between all sampling locations represented as a heatplot.

**
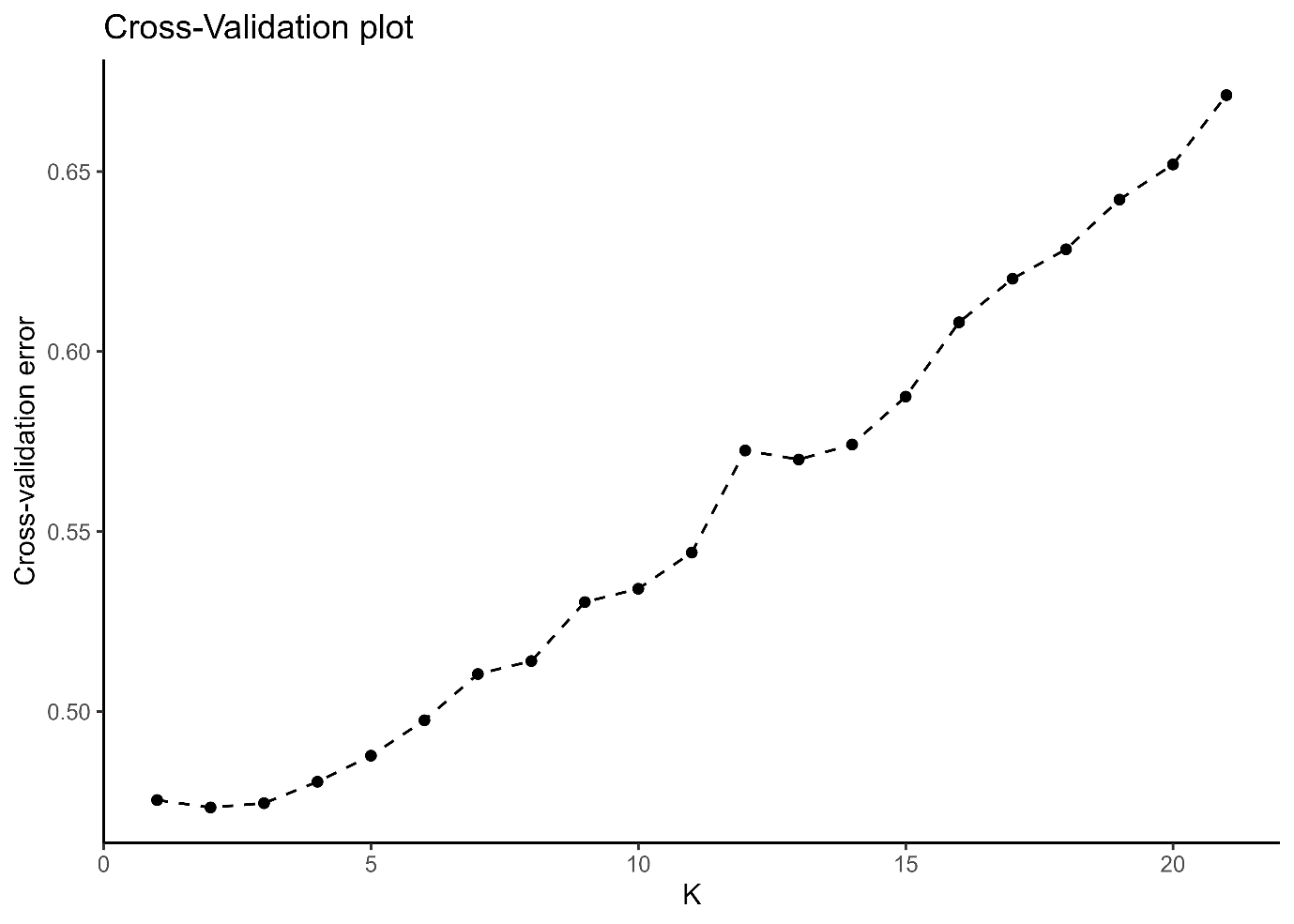
**

**Figure S2.** Cross-validation scores from ADMIXTURE analysis.

**
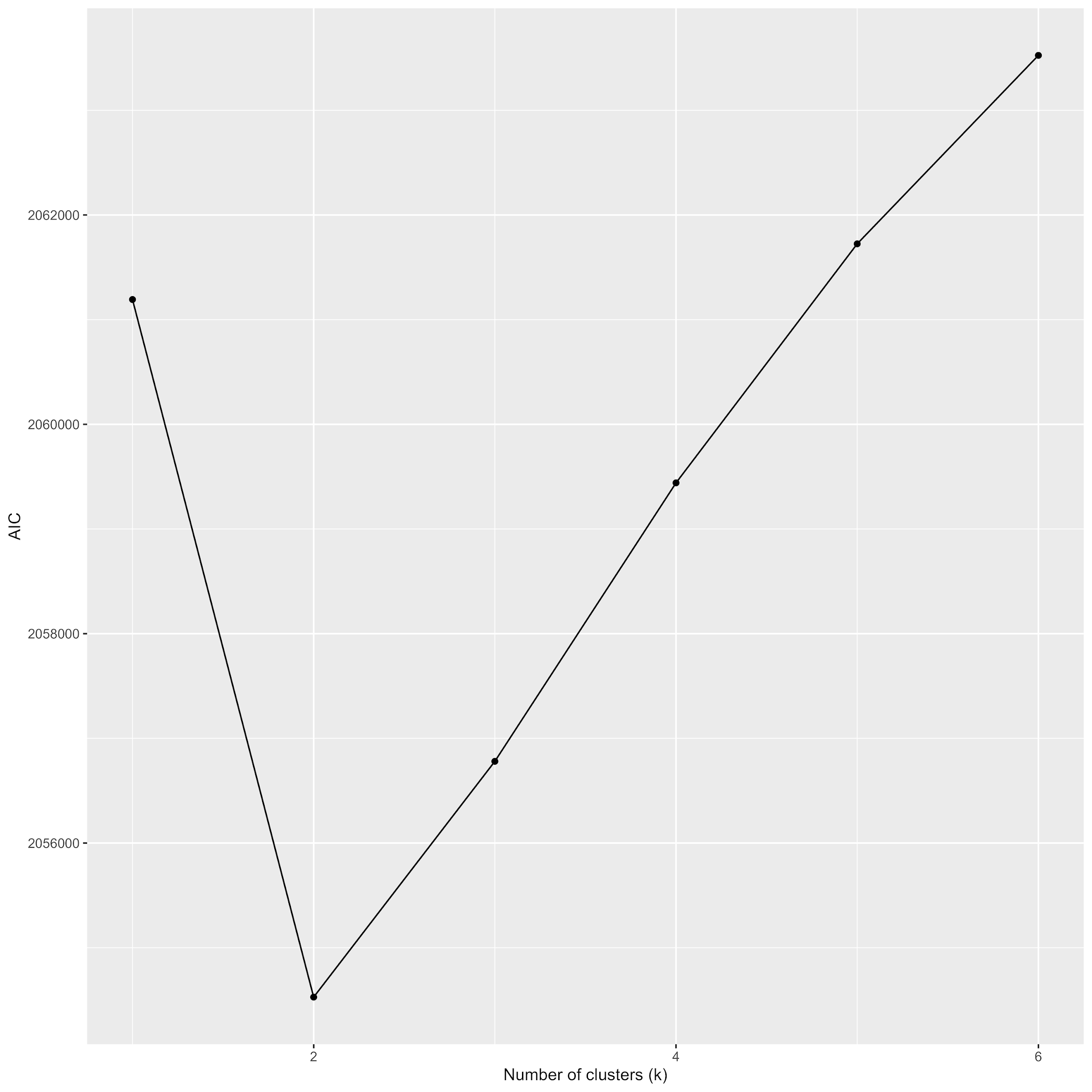
**

**Figure S3.** *K*-selection via the AICc method for Snapclust analysis

**
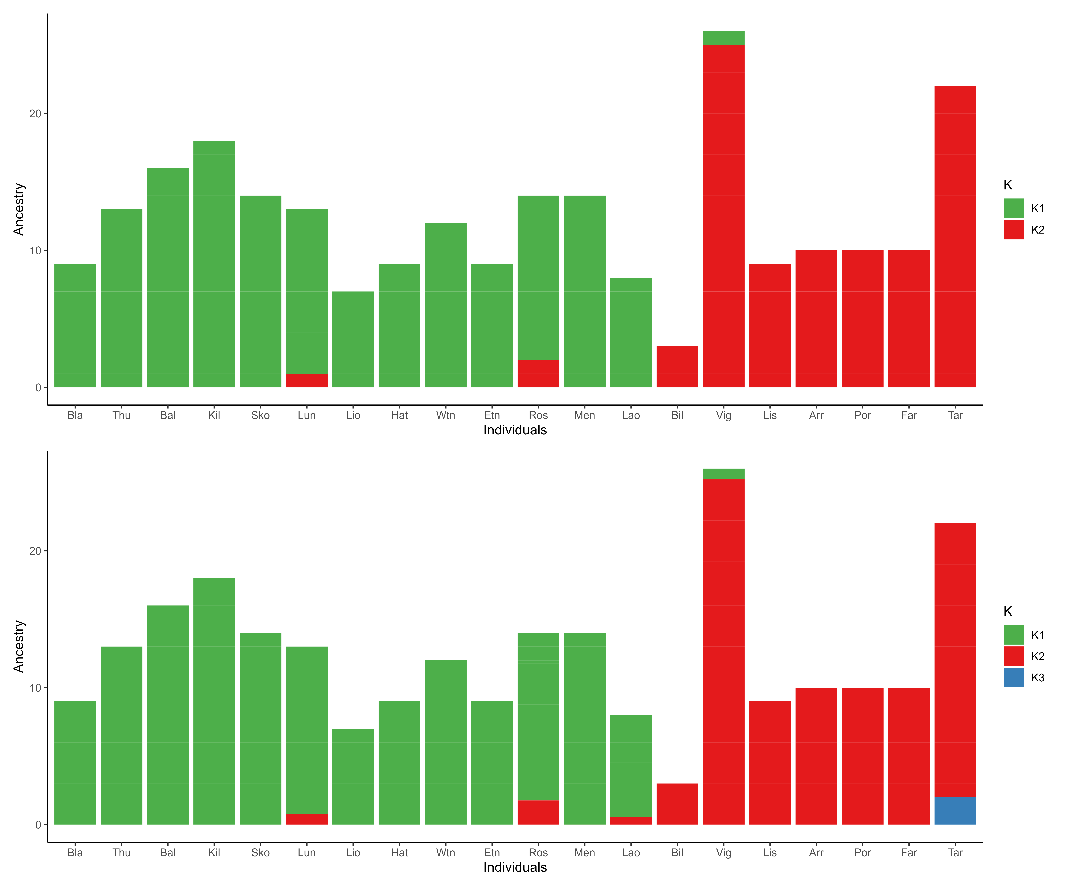
**

**Figure S4.** Visualisation of genetic clusters at *K* = 2 and *K* = 3 detected using Snapclust analysis.


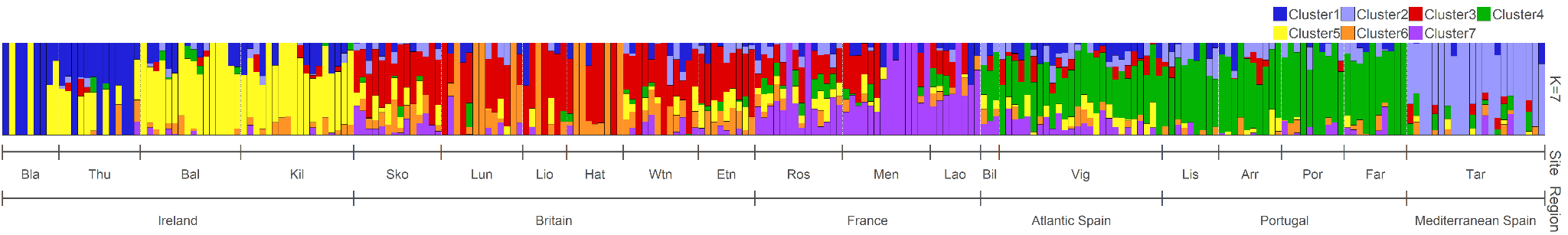


**Figure S5.** Visualisation of genetic clusters at *K* = 7 detected using ADMIXTURE analysis with no prior on location.

**
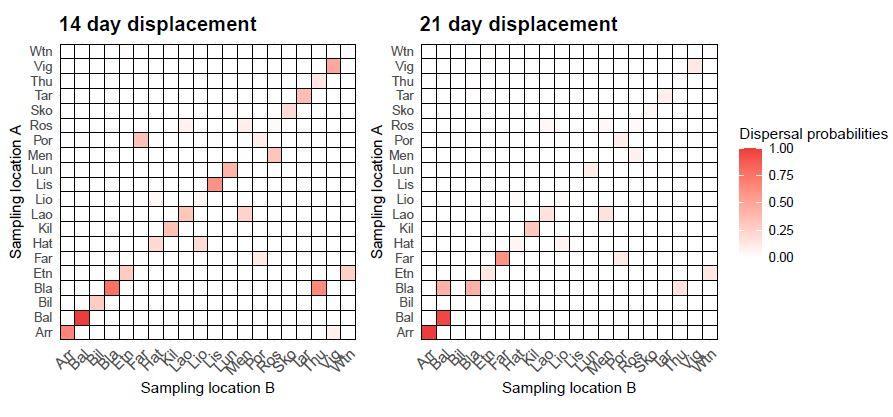
**

**Figure S6.** Connectivity matrix for a 14-day (left) and a 21-day PLD (right) simulation length. Dispersal probabilities represents the number of particles retained within the release site and the number of particles entering the 20 km buffer zone around each release site.

**
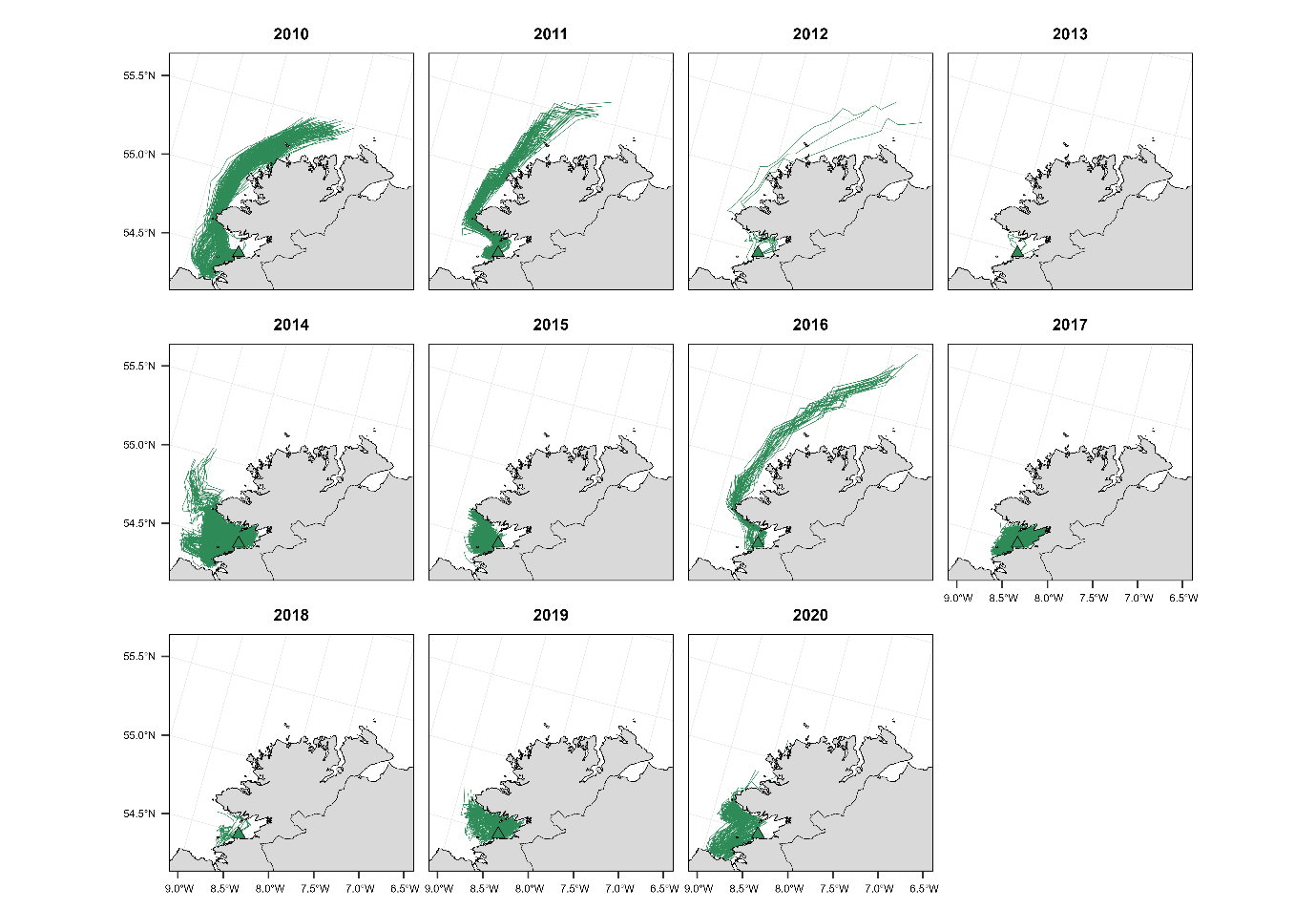
**

**Figure S7.** Yearly displacement trajectories under a 21-day PLD simulation for Thumb Rock, northwest Ireland.

**
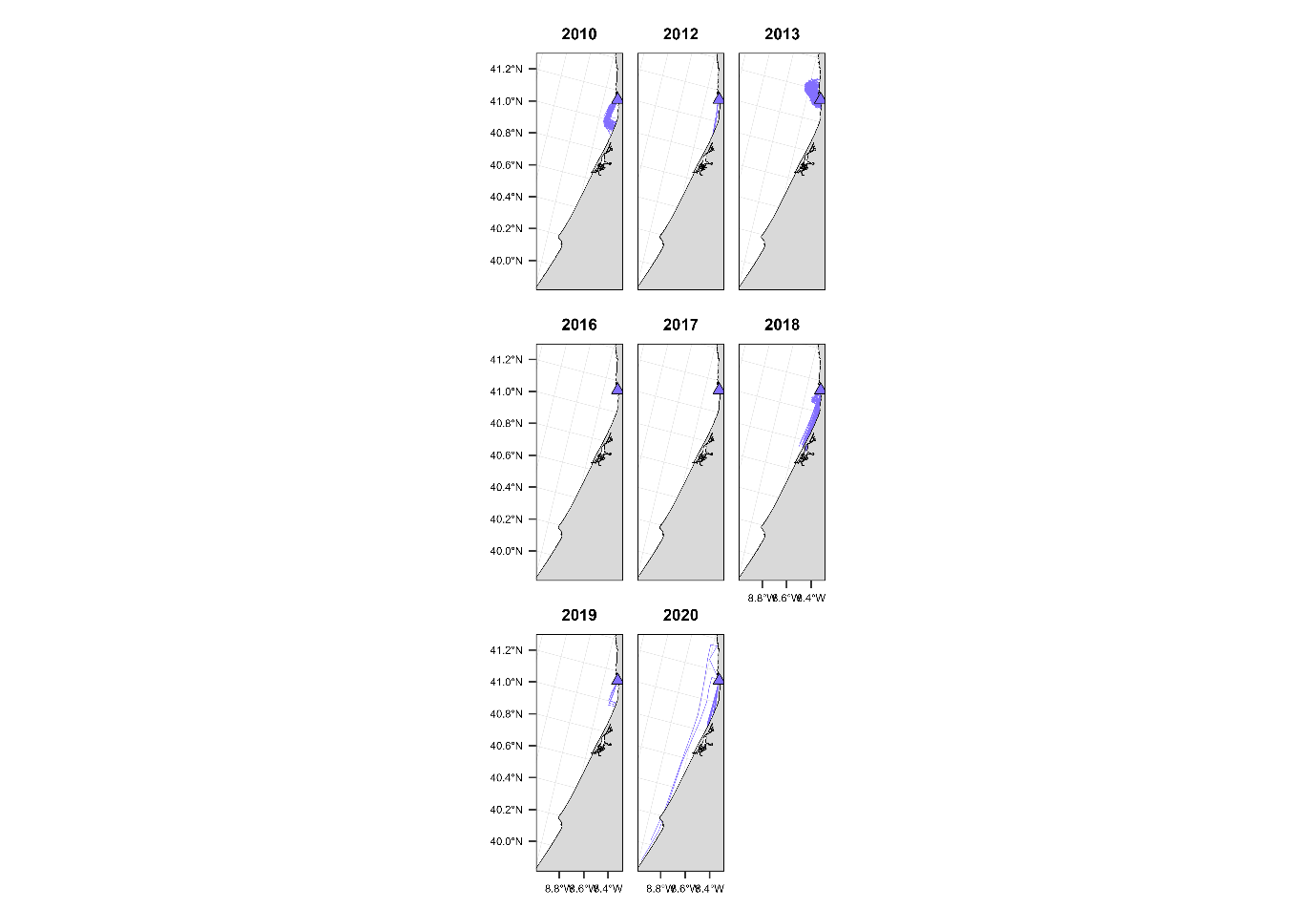
**

**Figure S8.** Yearly displacement trajectories under a 14-day PLD simulation for Arrabida, Portugal.

**
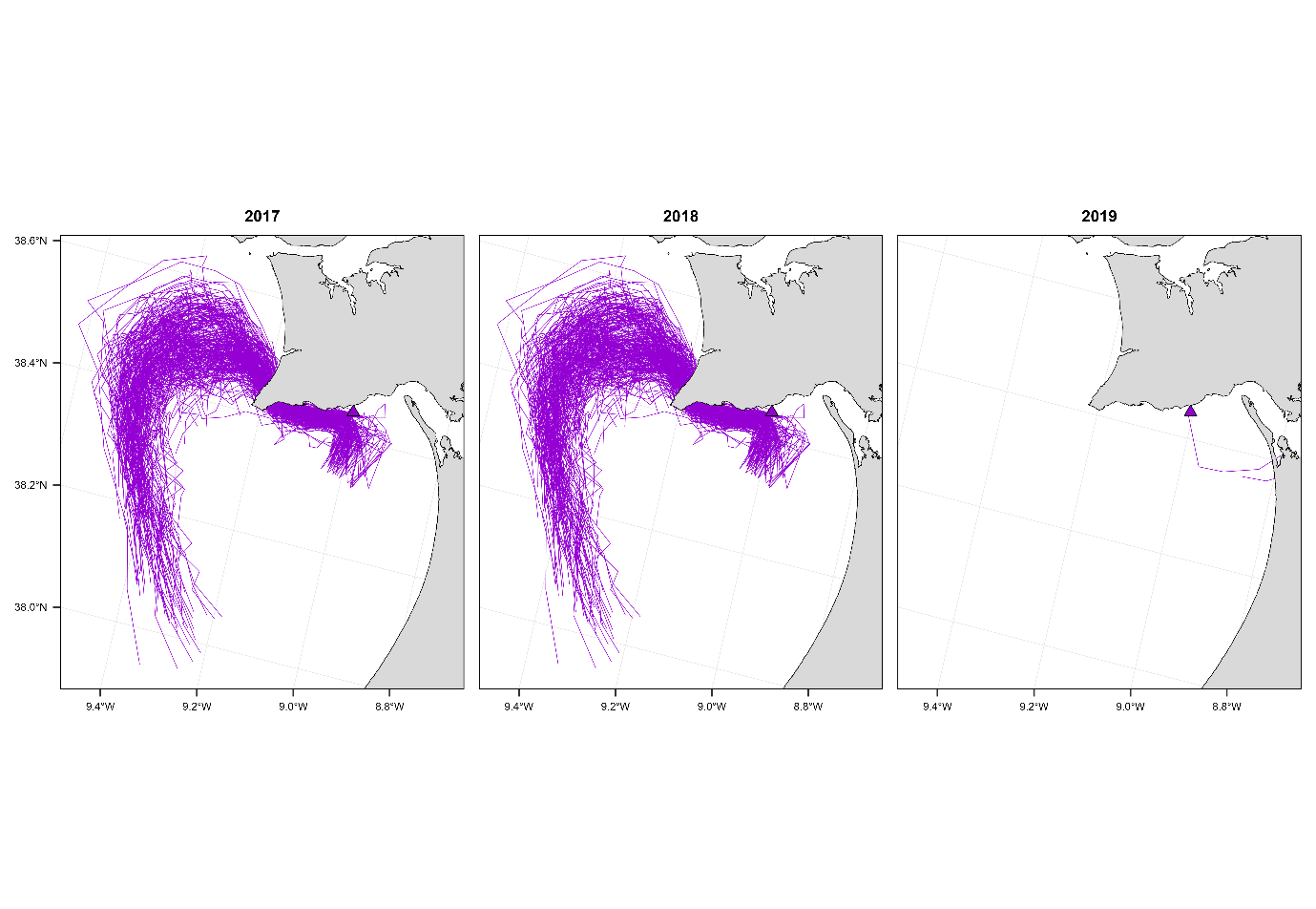
**

**Figure S9.** Yearly displacement trajectories under a 21-day PLD simulation for Lisbon, Portugal.

**
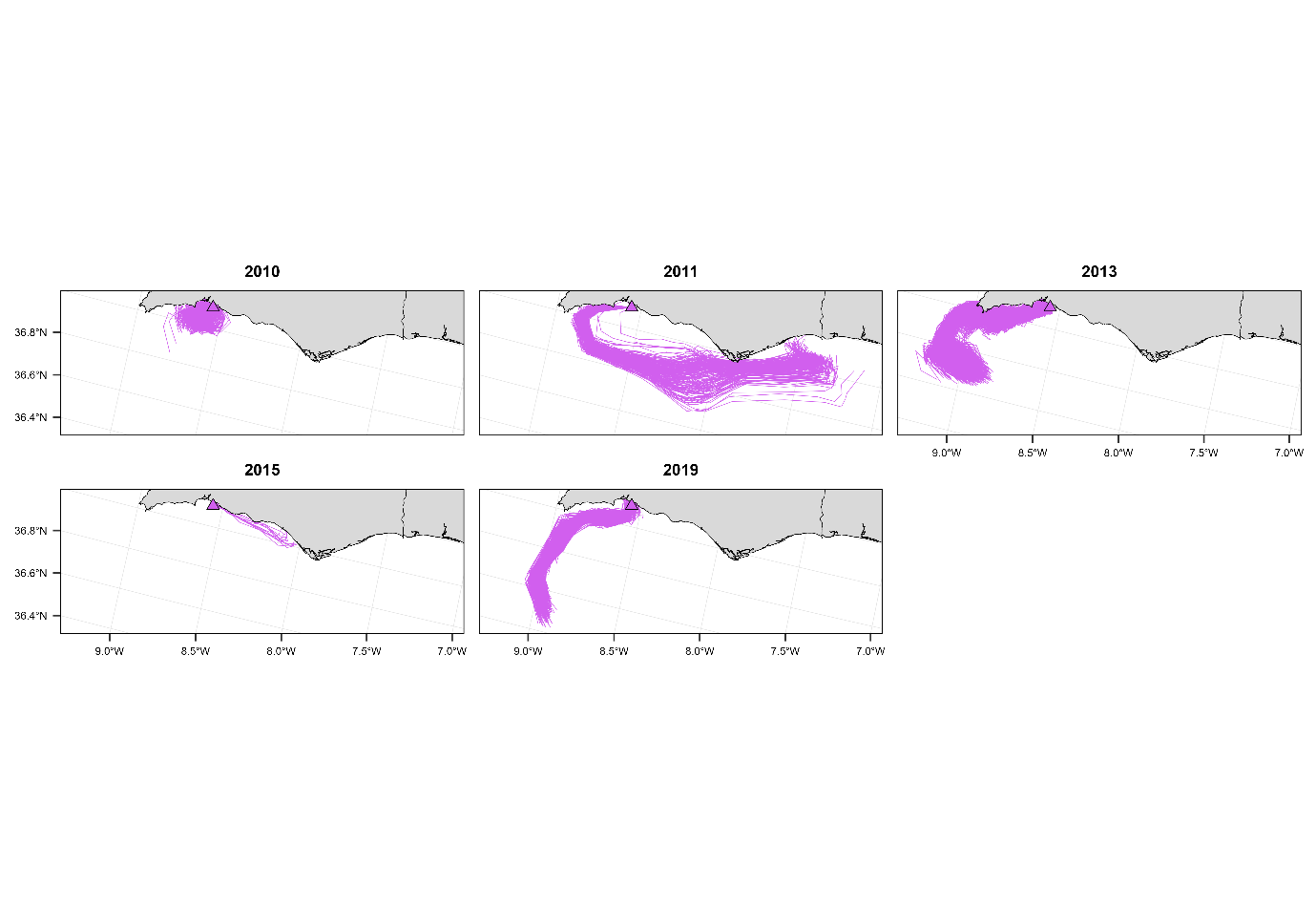
**

**Figure S10.** Yearly displacement trajectories under a 21-day PLD simulation for Portimão, Portugal.

**
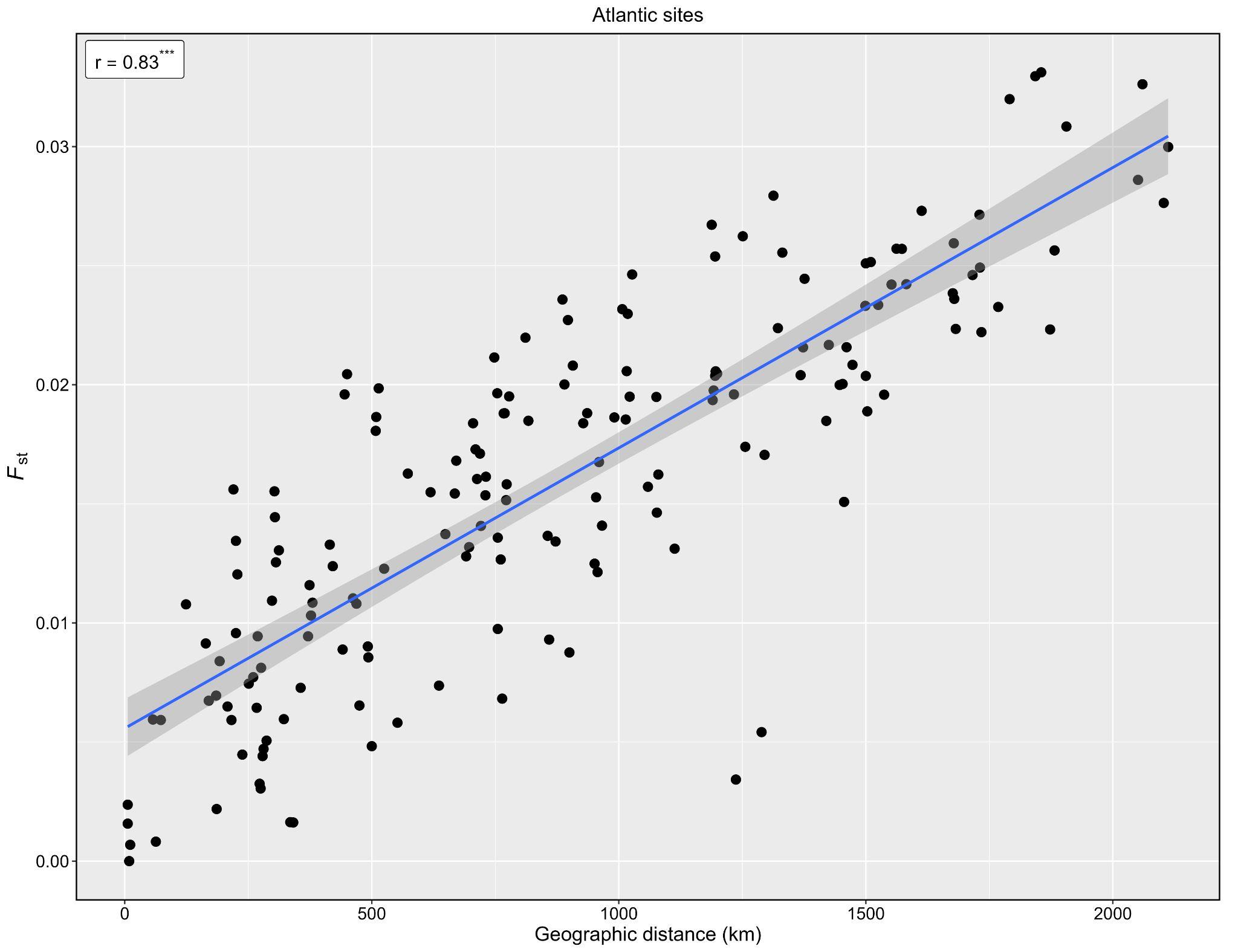
**

**Figure S11.** Isolation by distance (IBD) analysis of pairwise comparisons of geographic distances (km) and *F*_ST_ between all northeast Atlantic sampling sites.

**
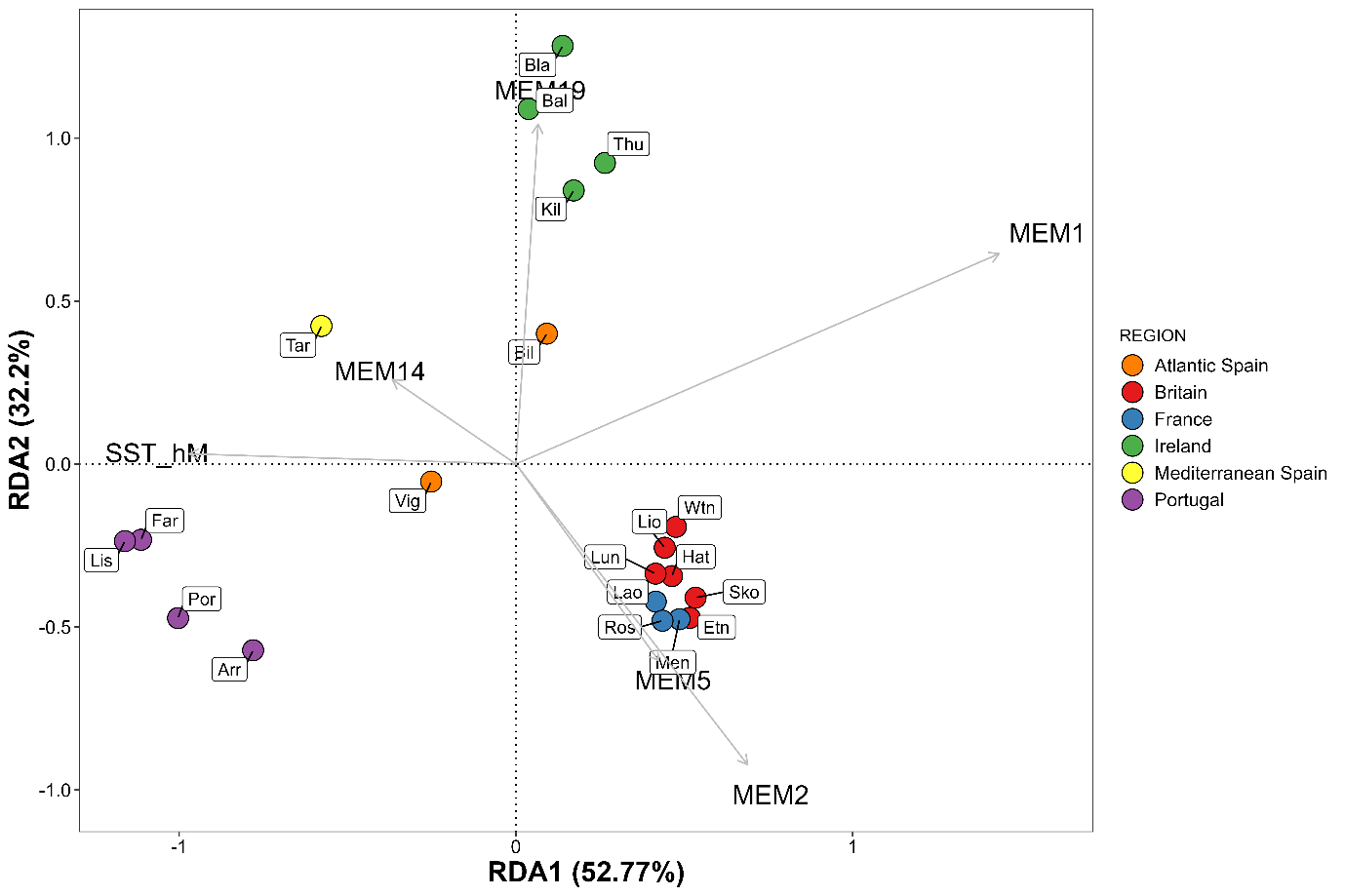
**

**Figure S12.** Redundancy analysis (RDA) performed on the 7,510 common neutral SNPs detected with OutFLANK and PCAdapt programs. RDA axis 1 represents 52.77% and RDA axis 2 represents 32.2% of the total genetic variation. Both axes were statistically significant. The RDA biplot shows the six significant explanatory db-MEM predictors: MEM1, MEM2, MEM5, MEM14 and MEM19 and environmental variable SST_hM (indicated by the arrow in grey). The direction of each arrow corresponds to the axis which the variable is related to. Each dot represents a sampling site and sites are colour-coded by region.

**
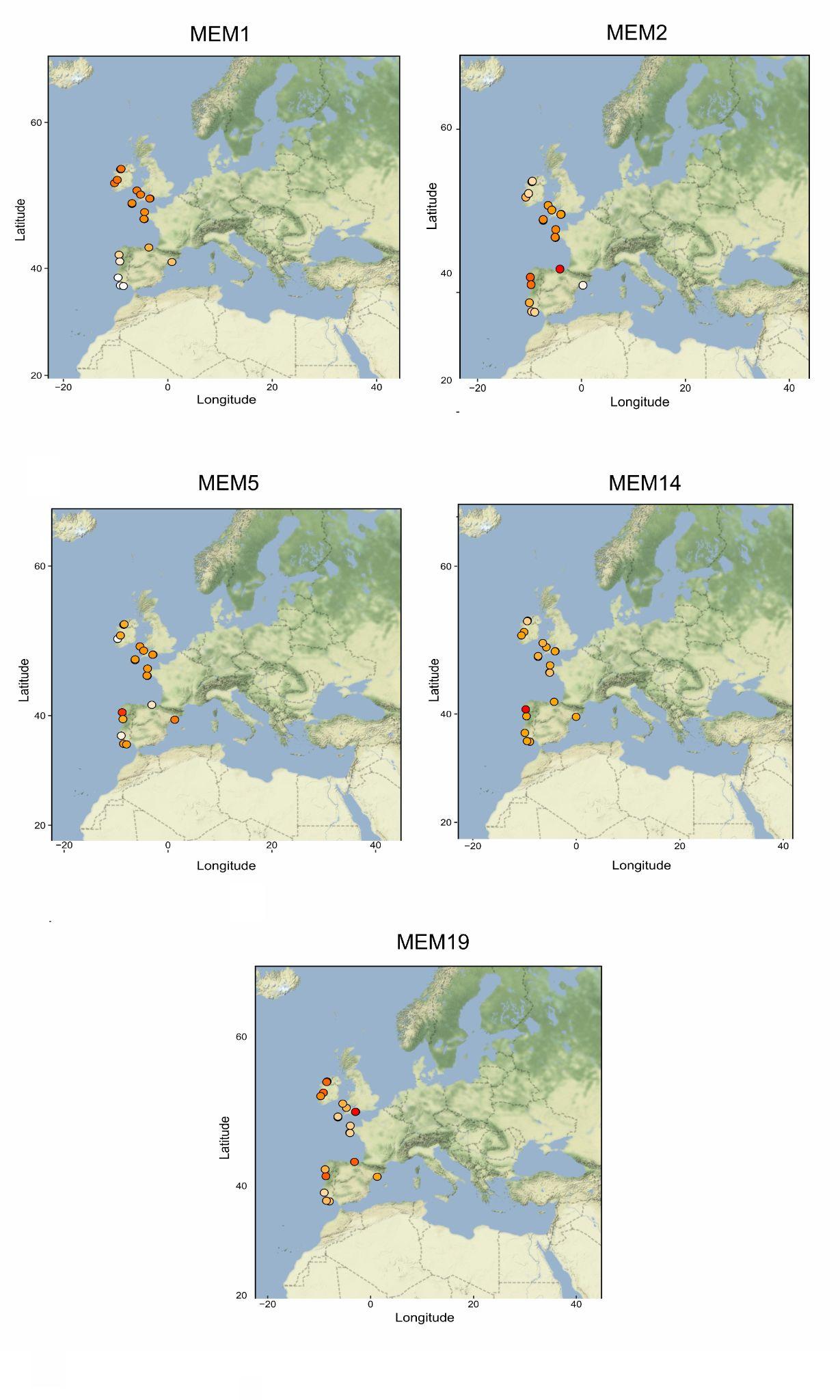
**

**Figure S13.** Visualisation of final five significant dbMEM predictor variables across the geographic extent of the study. MEM1 (top left) was highly correlated with mean annual sea surface temperature for the hottest month across the range of the study.

**Salting-out protocol for extracting genomic DNA from lobster pleopods/pink sea fan polyps**

**Materials**

Proteinase K (20 mg/ml)

RNase A (100 mg/ml)

1 % SDS cell lysis buffer (100mM Tris-Cl; 50 mM EDTA; 1 % SDS)

7.5 M ammonium acetate

0.5 M EDTA

Nuclease-free water

**Equipment**

TissueLyser and microbeads

1.5 ml microcentrifuge tubes

Sterile blue roll

Microcentrifuge and vortexer

**Protocol**

1. Remove sample from preservative and dap on sterile blue roll to remove excess ethanol.
2. Add samples (up to 30 mg) to 1.5 ml microcentrifuge tubes containing a microbead.
3. Homogenise samples by placing in a TissueLyser for 30 seconds at 30 Hz (repeat if necessary).

**Digestion**

1. Add the following to each tube:
   1. 350 µl 1 % SDS cell lysis buffer
   2. 42 µl 0.5 M EDTA
   3. 10 µl proteinase K.
2. Mix by vortexing and incubate at 65^o^C for 2.5 to 3 hours.
3. Add 2 µl RNase A and incubate on a thermomixer at 37^o^C for 30 minutes.

**Remove proteins and cellular debris**

1. Add 140 µl 7.5 M ammonium acetate to each tube. Mix by vortexing. Incubate at 4^o^C for 10 minutes.
2. Centrifuge at 12,000 g for 10 minutes.
3. Transfer supernatant to a new 1.5 ml microcentrifuge. Discard the previous tube.
4. Repeat steps 7-9.

**Precipitation of DNA**

1. Add 680 µl cold isopropanol (volume ratio 1:1). Mix by inverting gently 50 times. Centrifuge at 8000 g for 5 minutes.
2. Carefully discard the supernatant, avoiding contact with the pellet. Drain the tube by placing on sterile blue roll, taking care that the pellet remains in the tube.

**Washing of DNA**

1. Add 400 µl 70 % ethanol. Invert the tube several times to wash the DNA pellet. Centrifuge at 8000 g for 1 minute.
2. Carefully discard the supernatant, avoiding contact with the pellet. If a lot of supernatant remains, pulse centrifuge the tubes and discard the supernatant using a smaller pipette, again avoiding contact with the pellet.
3. Allow to air dry to 10-20 minutes. Non-contaminated pellets will turn more transparent as they dry. Avoid over-drying the DNA pellet, as the DNA will be difficult to dissolve.

**Rehydration of DNA**

1. Re-suspend dried pellets with 100 µl nuclease-free water. Invert tube to mix and spin down using centrifuge.
2. Incubate at room temperature for 30 minutes or incubate in the fridge overnight.
3. Briefly pulse centrifuge tubes and store at -20^o^C.

______________________________________________________________________
